# NuA4 histone acetyltransferase activity is required for H4 acetylation on a dosage-compensated monosomic chromosome that confers resistance to fungal toxins

**DOI:** 10.1186/s13072-017-0156-y

**Published:** 2017-10-23

**Authors:** Hironao Wakabayashi, Christopher Tucker, Gabor Bethlendy, Anatoliy Kravets, Stephen L. Welle, Michael Bulger, Jeffrey Hayes, Elena Rustchenko

**Affiliations:** 1 Department of Biochemistry and Biophysics, University of Rochester Medical Center, Rochester, NY USA; 2 Roche Diagnostics Corporation, Indianapolis, IN USA; 3 Parabase Genomics, Dorchester, MA USA; 4 Department of Medicine, University of Rochester Medical Center, Rochester, NY USA; 5 Department of Pediatrics, Center for Pediatric Biochemical Research, University of Rochester Medical Center, Rochester, NY USA

**Keywords:** *Candida albicans*, NuA4, H4 and H3 acetylation, Chromosome 5 monosomy

## Abstract

**Background:**

The major human fungal pathogen *Candida albicans* possesses a diploid genome, but responds to growth in challenging environments by employing chromosome aneuploidy as an adaptation mechanism. For example, we have shown that *C. albicans* adapts to growth on the toxic sugar l-sorbose by transitioning to a state in which one chromosome (chromosome 5, Ch5) becomes monosomic. Moreover, analysis showed that while expression of many genes on the monosomic Ch5 is altered in accordance with the chromosome ploidy, expression of a large fraction of genes is increased to the normal diploid level, presumably compensating for gene dose.

**Results:**

In order to understand the mechanism of the apparent dosage compensation, we now report genome-wide ChIP-microarray assays for a sorbose-resistant strain harboring a monosomic Ch5. These data show a significant chromosome-wide elevation in histone H4 acetylation on the mCh5, but not on any other chromosome. Importantly, strains lacking subunits of the NuA4 H4 histone acetyltransferase complex, orthologous to a complex previously shown in *Drosophila* to be associated with a similar gene dosage compensation mechanism, did not show an increase in H4 acetylation. Moreover, loss of NuA4 subunits severely compromised the adaptation to growth on sorbose.

**Conclusions:**

Our results are consistent with a model wherein chromosome-wide elevation of H4 acetylation mediated by the NuA4 complex plays a role in increasing gene expression in compensation for gene dose and adaption to growth in a toxic environment.

**Electronic supplementary material:**

The online version of this article (doi:10.1186/s13072-017-0156-y) contains supplementary material, which is available to authorized users.

## Background

*Candida albicans* is an opportunistic fungal pathogen that is part of the normal microbial community of the digestive tract and genitalia of humans. *C. albicans* does no harm to the healthy host; however, it causes life-threatening infections in severely immunocompromised patients. *C. albicans* normally possesses a diploid genome organized in eight pairs of chromosomes, but uses reversible loss or gain of an entire chromosome or a large part of chromosomes to survive in toxic environments that would otherwise kill cells or prevent their propagation [reviewed in 1, 2]. For example, the loss of one chromosome 5 (Ch5) or duplication of the right arm of Ch5 confers laboratory resistance to the anti-fungal caspofungin [3, 4]. The loss of one Ch5 also confers laboratory resistance to the anti-fungal flucytosine, as well as resistance to toxic sugar l-sorbose, which kills *C. albicans* in a manner similar to caspofungin or other frontline drugs from the echinocandin class [reviewed in 3]. In addition, changes in Ch5 ploidy are frequently found in fluconazole-resistant clinical isolates of *C. albicans* [reviewed in 5, 6]. In light of data pointing to the importance of Ch5 in *C. albicans* drug resistance, there is a growing need to better understand the control of Ch5 ploidy and regulation of genes on this chromosome.

**Fig. 1:**
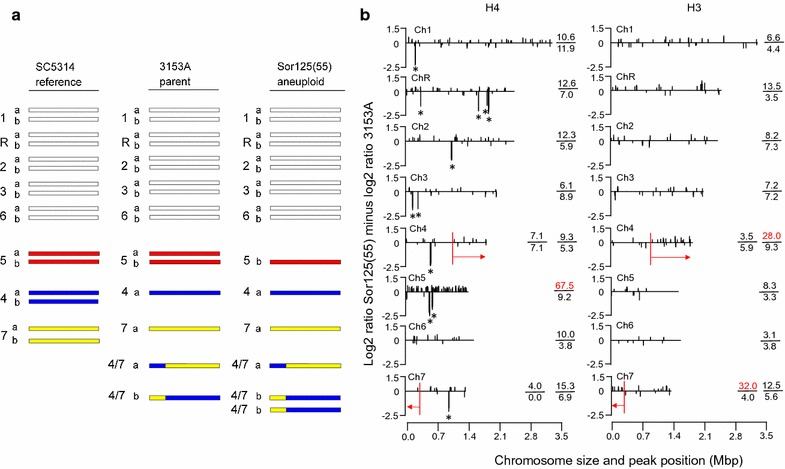
Schematic chromosome patterns and ChIP-Chip results. **a** Horizontal bars represent the individual chromosomes of the parental strain 3153A, its aneuploid derivative Sor125(55), and the reference strain SC5314, as indicated. Chromosomes are designated from 1 to 7 and R, on the left, as their sizes decrease from top to bottom. For the chromosome sizes, see [8]. ChR refers to the chromosome containing a cluster of tandemly repeated rDNA units. Homologous chromosomes are indicated with “*a*” and “*b.*” [Adopted from 8]. **b** Graphic presentation of ChIP-Chip results showing acetylation of histone H4 and histone H3, as indicated, on the chromosomes of the mutant Sor125(55), compared to the parental strain 3153A. Each chromosome is presented with a single graph. The *X*-axis indicates the position of the probes on each chromosome in the reference strain SC5314, as annotated in genomic assambly 21 in CGD. The *Y*-axis shows the averaged log2 ratio Sor125(55) minus log2 ratio 3153A (see “Methods”). The horizontal red arrows on Ch 4 and Ch7 indicate the trisomic regions due to duplication of Ch4/7b (see Fig. 1a). The numbers on the right indicate the density of positive (top) and negative (bottom) peaks of acetylation on each chromosome, while the second values on the right for Ch4 and Ch7 indicate the density of positive and negative peaks within the trisomic regions. Asterisks indicate large negative peaks denoting apparent loss of H4 acetylation in Sor125(55)

Previous studies of changes in transcription of Ch5 genes associated with the loss of one Ch5 in a mutant [Sor125(55)] adapted to growth on the toxic sugar sorbose (Sou^+^) revealed expression of many genes on the monosomic Ch5 decreased twofold, conforming to the loss of DNA copy number. However, surprisingly, expression of ~ 40% of gene transcripts from monosomic Ch5 corresponded to the normal disomic or near disomic levels [7]. In addition to monosomy of Ch5, this mutant acquired duplication of a chimeric Ch4/7b, resulting in trisomy of this chromosome (Fig. 1a), and facilitating the Sou^+^ phenotype [8]. Similar to Ch5, our data show that while expression of many genes on the trisomic Ch4/7b increased 1.5-fold, as expected from gene copy number, many other genes were expressed at the normal disomic or near disomic levels (C. Tucker and E. Rustchenko, unpublished observation). These observations led us to propose that in *C. albicans*, transcriptional compensation for gene dose serves to facilitate the formation and maintenance of aneuploid chromosome states that are required for survival in adverse environments [7]. We have previously reported multiple genes encoding negative regulators of laboratory resistance to echinocandins or sorbose that are scattered across Ch5 [9]. While the final number of regulatory genes is yet to be determined, some are involved in cell wall biosynthesis, while others important to the Sou^+^ and drug susceptibility phenotypes are subjects of antisense regulation [9–11]. However, despite these advancements, little is known about the formation and maintenance of the monosomic Ch5 state.

The regulation of gene expression on the monosomic Ch5 in *C. albicans* is possibly analogous to the well-described and essential dosage compensation found on the single male X chromosome in *Drosophila melanogaster*. In this organism, compensation requires histone H4 acetylation mediated by the male-specific lethal (MSL) complex, which includes the histone acetyltransferase (HAT) subunit MOF [12]. MSL acetylation of H4 is associated with twofold upregulation of almost all genes on the single X chromosome. The orthologous complex in *C. albicans* is termed the nucleosome acetyltransferase of H4 (NuA4) complex, in which the Esa1p subunit harbors the catalytic activity [13]. The NuA4 complex is evolutionarily conserved from yeast to humans and is known to be a key regulator of transcription [14, 15]. In *Saccharomyces cerevisiae*, NuA4 is responsible for the acetylation of nucleosomal histone H4 at lysine K5, K8, and K12 and, to a lesser extent, at K16. In *C. albicans*, NuA4 contributes mainly to acetylation of H4 K5 and H4 K12, whereas the SAS HAT complex contributes to acetylation of H4 K16 [16]. In support, deletion of a gene encoding the NuA4 catalytic subunit Esa1p in *C. albicans* significantly diminished acetylation of H4 K5 and H4 K12 [16]. Also, deleting Nbn1p, which comprises part of the NuA4 catalytic module, diminished overall H4 acetylation [17].

Considering the well-characterized model of dosage compensation mechanisms operative on the single X chromosome of *D. melanogaster,* we wished to determine whether elevated H4 acetylation is similarly associated with the aforementioned widespread transcriptional upregulation found on the monosomic Ch5 in the *C. albicans* sorbose-resistant mutant [7]. Indeed, we found increased H4 acetylation exists across the monosomic Ch5, while, in contrast, increased H3 acetylation is found within the trisomic Ch4/7b region. Importantly, we show that NuA4 subunits are required for elevated H4 acetylation in strains bearing a monosomic Ch5 and for efficient adaption to growth of *C. albicans* in the presence of the toxic sorbose.

## Results

### Acetylation of H4 and H3 on aneuploid chromosomes in *C. albicans*

We first asked whether histone H4 acetylation patterns across the genome are altered in the well-characterized sorbose-resistant strain Sor125(55) containing a monosomic Ch5 and a trisomic Ch4/7b, compared to the normal diploid parental strain (Fig. 1a). We performed chromatin immunoprecipitation on chip (ChIP-Chip) using a pan-H4-acetylation antibody with hybridization to our custom tiling arrays (see “Methods”). Analysis of the data shows sporadic changes in acetylation genome wide, with both increases and decreases apparent at many loci (Fig. 1b, note that the majority of loci have changes that do not exceed a threshold and are not plotted). However, we observed a generalized and much larger fivefold to tenfold increase in H4 acetylation on the monosomic Ch5 than that found on all other chromosomes. All chromosomes showed similar amounts of decreased H4 acetylation.

We then asked whether the greatly elevated level of histone acetylation we observe on the monosomic Ch5 is specific to histone H4 or a more generalized feature of nucleosomes in the mutant strain by examining genome-wide acetylation of histone H3, which is also commonly increased in association with transcription. We performed ChIP-Chip using an antibody specific for H3 acetylated at the transcription-related sites K9 and K14 and found that H3 acetylation on Ch5 appeared similar to that found on all other disomic chromosomes, exhibiting a sporadic pattern of increases and decreases at various loci in the mutant compared to the parental strain. In contrast, we detected approximately a twofold to tenfold overall increase in H3 acetylation localized to the trisomic Ch4/7b region of the genome (Fig. 1b).

We also noticed a number of large negative peaks in the H4 acetylation plot, indicating loci exhibiting significant decreases in this modification in the mutant compared to parental cells (marked with asterisks in Fig. 1b, note that many of these peaks are too closely positioned to be distinguished in this figure, but are listed in Additional file 1: Table S1). There were 43 such peaks scattered throughout the genome, associated with 27 open reading frames (ORFs) based on the criteria of being positioned either within 1 kb of an ORF or within of an ORF. The function of the majority of the peak-associated genes is not known, thus understanding the phenotypic outcome associated with these changes in acetylation awaits further analysis.

### The NuA4 complex plays a role in elevated H4 acetylation on the monosomic Ch5

As described above, elevated histone acetylation associated with dosage compensation of the single X chromosome in male flies is due to the HAT MOF that acetylates histone H4 [12]. To determine whether the orthologous NuA4 complex in *C. albicans* is involved in the large increase in acetylation observed across Ch5 in sorbose-resistant [Sor125(55)] cells, we initially employed three *C. albicans* strains, each with the NuA4 complex disrupted. Specifically, we employed strain Nbn1 (*nbn1* −/−) in which both alleles of the *NBN1* (orf19.878) gene encoding the Nbn1p subunit of the catalytic NuA4 HAT module are deleted. We also used the strain Eaf3 (*eaf3* −/−), in which both alleles of orf19.2660, encoding Eaf3p, an ortholog of yeast *EAF3* within the targeting module of the *S. cerevisiae* NuA4 complex, are deleted. Finally, we employed strain Esa1 (*esa1* *−/+*), in which one allele of the essential *ESA1* (orf19.5416) gene encoding the catalytic subunit of the NuA4 complex is disrupted (Table 1). PCR and Southern blot analyses confirmed disruption of one *ESA1* allele and deletion of both alleles of *NBN1* and *EAF3* in these strains (data not shown).

**Table 1:** *Candida albicans* strains used in this study

Strain	Genotype	Source
BWP17	*ura3*-*∆::imm434/ura3*-*∆::imm434*, *arg4*-*∆::hisG/arg*-*∆::hisG*, *his1*-*∆::hisG/his1*-*∆::hisG*	Tag-Module collection
Esa1	Same as BWP17, but *esa1*-∆/*ESA1*	Same as above
DAY286	Same as BWP17, but *ura3*-*∆::imm434/ura3*-*∆::imm434, ARG4::URA3::arg4*-*∆::hisG/arg*-*∆::hisG, his1*-*∆::hisG/his1*-*∆::hisG*	FGSC^a^
DAY286-1	Same as DAY286, but Ch5 monosomy	This study
Nbn1	Same as DAY286, but *nbn1*-∆/*nbn1*-∆	FGSC
Nbn1-1	Same as Nbn1, but Ch5 monosomy	This study
Nbn1-2	Same as Nbn1, but Ch5 monosomy	This study
Nbn1-3	Same as Nbn1, but Ch5 monosomy	This study
Eaf3	Same as DAY286, but *eaf3*-∆/*eaf3*-∆	FGSC
Eaf3-1	Same as Eaf3, but Ch5 monosomy	This study
3153A	Laboratory strain, normal diploid	[8, 27]
Sor125(55)		
a.k.a. Sor55	Same as 3153A, but Ch5 monosomy, *MTLα*, and Ch4/7b trisomy^b^	[8, 27]

^a^Fungal Genomic Strains Center

^b^Both Sor125(55) and its parental strain, 3153A, harbor two hybrid chromosomes resulting from a reciprocal exchange between a Ch4 and a Ch7 of the reference strain SC5314 [8], which we term Ch4/7a and Ch4/7b. In Sor125(55), Ch4/7b is duplicated; therefore, genes on this hybrid chromosome, together with the corresponding portions of the intact Ch4 and Ch7, have a copy number of three [8]

**Fig. 2:**
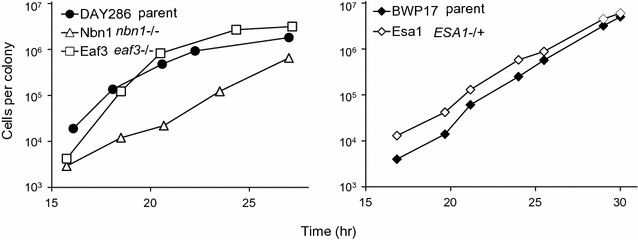
Growth curves of strains with a disrupted NuA4 complex. Shown are Nbn1 (*nbn1* −/−) and Eaf3 (*eaf3* −/−) versus parental DAY286 (left) and Esa1 (*ESA1* *−/+*) versus parental BWP17 (right), as indicated. See Table 1 for the strains’ relationship and genotype

We first tested how loss of the NuA4 subunits affected the growth of Esa1, Nbn1 or Eaf3 cells, as compared to their parent strains. We spread approximately 3000 colony-forming units (cfu) of each strain on plates with YPD universal rich medium and, when micro-colonies appeared, measured number of cells in colonies in approximately 4-h intervals. We found that the loss of Nbn1p from the catalytic module of the NuA4 complex greatly affected the growth, whereas the loss of Eaf3p from the targeting module or disruption of one copy of *ESA1* encoding the NuA4 catalytic subunit had little effect on growth (Fig. 2). The Esa1 strain was not included in further experiments because the effect of disruption of a single copy of *ESA1* might not result in NuA4 insufficiency [for example, see 9].

We next assessed whether disruptions of the NuA4 complex altered histone acetylation levels upon transition of Nbn1 or Eaf3 cells from disomic to monosomic state of Ch5. We generated monosomic Ch5 mutants from the NuA4^+^ parental strain DAY286 and the NuA4 deletion mutants Nbn1 and Eaf3 (Table 1) using our well-established method of plating cells on l-sorbose medium [18] (see “Methods”). We confirmed loss of one Ch5 in derived mutants by PCR with primers for *MTL* loci, residing on Ch5 [7]. We also examined the chromosome banding pattern in one mutant from each parental strain by pulse-field gel electrophoresis (PFGE) (Additional file 2: Figure S1). PFGE analysis showed that ChR carrying tandem repeats of ribosomal DNA (rDNA) units exhibited some instability in the Nbn1 monosomic Ch5 mutant as expected, since changes of ChR lengths frequently occur due to changes in the number of rDNA units in the cluster of tandemly assembled rDNA units [19]. Otherwise, chromosome banding patterns did not show any other alterations in mutant strains other than monosomy of Ch5, common to all mutants. It is important to note that adaptation of *C. albicans* to growth on sorbose as the sole source of carbon almost exclusively involves a transition to a monosomic Ch5 state without other chromosome alterations, in multiple genetic backgrounds (> 95% incidence) [3, reviewed in 1, 2, 20], and this method is now used by many laboratories.

**Fig. 3:**
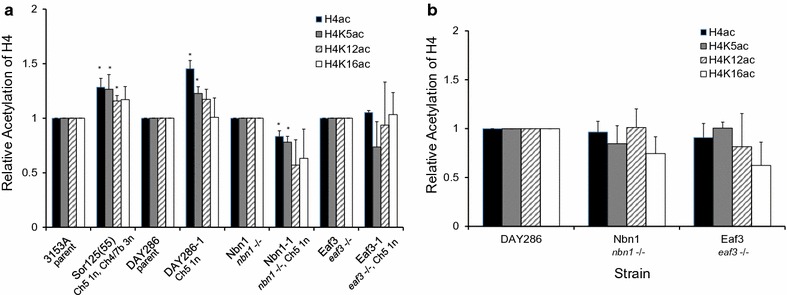
Increase in H4 acetylation associated with adaptation of monosomic Ch5 state requires an intact NuA4 complex. **a** Comparison of total H4 acetylation in parental *C. albicans* strains and monosomic Ch5 strains derived as described in the tex. Shown is total H4 acetylation in: (i) the parental strain (3153A) and the monosomic Ch5 mutant Sor125(55); (ii) the parental strain DAY286 and the monosomic Ch5 mutant DAY286-1; (iii) parental strain Nbn1 (*nbn* −/−) and the monosomic Ch5 mutant Nbn1-1; and (iv) the parental stran Eaf3 (*eaf* −/−) and the monosomic Ch5 mutant Eaf3-1. **b** Total H4 acetylation in deletion mutants Nbn1 and Eaf3 compared to their parental strain DAY286. H4 acetylation was determined by Western blot with the indicated antibodies and results normalized to the parental strain response for each set (see text). Data are averaged from three independent experiments. Asterisks indicate statistical significance of the difference in acetylation between mutants and parentals with *p* value < 0.05 calculated according to Student’s *t* test

Having established the monosomic Ch5 strains in the desired parental and deletion backgrounds, we next employed Western blot analysis with either pan-acetyl H4 antibody or antibodies, specific for acetylation at H4 K5, H4 K12, or H4 K16 to compare acetylation in these strains. Importantly, we found a significant increase in total acetylation of H4 in the Sor125(55) strain that harbors a monosomic Ch5, compared to the parent 3153A (Fig. 3a, also see Additional file 3: Figure S2), consistent with our ChIP analysis (Fig. 1b). Likewise, we observed a significant total increase in H4 acetylation in the newly generated monosomic Ch5 mutant DAY286-1 versus its parent DAY286 (Fig. 3a, also see Additional file 3: Figure S2). Increased acetylation was also detectable with antibodies specific for H4 K5ac or K12ac [Sor125(55)], or H4 K5ac (DAY286-1). These data are in good agreement with our genome-wide ChIP analyses and indicate that the large elevation of H4 acetylation specific to Ch5 in strains monosomic for Ch5 can be observed in blots of histones isolated from whole cells.

We next compared H4 acetylation in parental cells lacking subunits from the NuA4 HAT and strains harboring a monosomic Ch5 derived from these cells. In a strain lacking the Nbn1 subunit from the catalytic module of NuA4, we found no difference in overall H4 acetylation compared to the parental DAY286 (Fig. 3b, black bar) and no significant difference was detected with antibodies specific for single acetylation events in the H4 tail (Fig. 3b, gray, hatched and white bars). Importantly, in contrast to Ch5 monosomic strains with a fully functional NuA4 complex, we found no increase in H4 acetylation associated with transition of the Nbn1 deletion strain to a monosomic Ch5 state (Fig. 3a, Nbn1-1, black bars). See results for two more independent Ch5 monosomic derivatives of Nbn1 in Additional file 4: Figure S3. The result was similar whether comparing pan-acetylation of H4 or specific acetylation at individual H4 lysines (Fig. 3a, Nbn1-1, gray, hatched, and white bars). Likewise, in cells lacking the Eaf3p subunit from the targeting module of NuA4, no increase in overall acetylation was observed upon transition from a disomic to monosomic Ch5 state (Fig. 3a, compare Eaf3 and Eaf3-1). Our results indicate that the elevated H4 acetylation observed in mutants with monosomic Ch5 is due to the activity of the NuA4 complex.

### Role of the NuA4 complex in formation of the monosomic Ch5

**Fig. 4:**
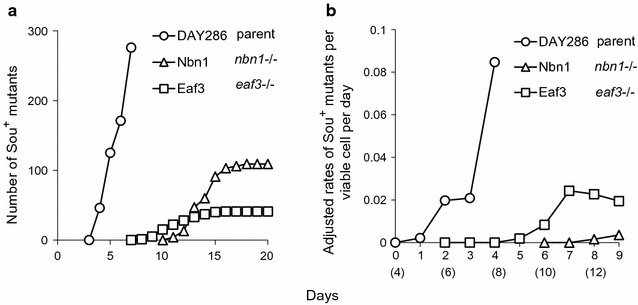
Daily accumulation of Sou^+^ colonies (**a**) and adjusted rates of generation of Sou^+^ mutants per viable cell per day (**b**). Shown are deletion strains Nbn1 (*nbn1* −/−) and Eaf3 (*eaf3* −/−), as well as their parental strain DAY286, as indicated. Note that in (**b**), both the deduced time of formation of the mutations (top number) and the time of the appearance of the corresponding Sou^+^ colonies (bottom number, in parentheses) are presented. See Results section for more explanations. Note the delayed and diminished production of Sou^+^ colonies and diminished mutant rates by Nbn1 and Eaf3

To determine whether the NuA4 complex has a role in formation of the monosomic Ch5 state, we determined whether the rates of production of Sou^+^ cells monosomic for Ch5 are affected by deletion of Nbn1 or Eaf3 subunits compared to the parental strain DAY286. The experiments were conducted, as previously reported [18]. Production of Sou^+^ colonies representing Sou^+^ mutants was observed for each strain by spreading from 2 × 10^5^ to 6 × 10^5^ of the Sou^−^ cells on l-sorbose plates in three independent experiments. The appearance of Sou^+^ colonies was recorded daily and is presented as an averaged accumulation curve (Fig. 4a). Survival of Sou^−^ cells was assessed from sorbose plates by daily transfer of entire agar disks to YPD plates, incubation for 3 days, and counting the number of grown colonies [see 18 for more details]. The daily survival of Sou^−^ cells of each strain is presented in Additional file 5: Figure S4. The daily rate of production of Sou^+^ mutants was calculated by dividing the number of Sou^+^ colonies appearing daily by number of cells that were viable 4 days earlier, the predicted day of the mutational events leading to transformation (Fig. 4b). [For example, we considered that mutational events that occurred in parental DAY286 cells on day 0 would result in appearance of visible Sou^+^ colonies on day 4 (Fig. 4a)]. We found greatly diminished rates of Sou^+^ mutants’ production with strains Nbn1 and Eaf3 lacking NuA4 subunits versus DAY286 with a normal NuA4 complex. This result shows that efficient adaptation to sorbose by formation of the monosomic Ch5 depends on the NuA4 complex.

## Discussion

In this work, we show that a chromosome-wide increase in acetylation of histone H4 occurs on the single Ch5 of the *C. albicans* sorbose-resistant mutant Sor125(55), on which ~ 40% of genes are upregulated to disomic or near disomic levels, thus compensating for decreased gene dosage. Such an increase in H4 acetylation seems to be a general attribute of the monosomic Ch5 state, as it also occurred on the single Ch5 in the mutant DAY286-1, which has a different genetic background. Increased H4 acetylation, however, does not occur on the trisomic Ch4/7b in the Sor125(55) mutant, on which multiple genes are downregulated to disomic or near disomic levels, again compensating for increased gene dosage (E. Rustchenko, unpublished observation). Instead, a chromosome-wide increase in acetylation of histone H3 occurs on the trisomic Ch4/7b, but does not occur on the monosomic Ch5 or any disomic chromosome. Thus, unique epigenetic signals occur on chromosomes with different aneuploidies, consistent with distinct transcriptional responses. We provide evidence that the bulk of the increased acetylation of histone H4 is due to activity of the NuA4 HAT complex, orthologous to the enzyme complex previously implicated in dosage compensation on the single male X chromosome in *Drosophila.* While the enzymes responsible for the observed increase in H3 acetylation have yet to be identified, previous work in *C. albicans* suggests that the Rtt109 or SAGA/ADA complexes might be responsible [21, 22].

Interestingly, disruption of a single allele of the essential *ESA1* gene encoding the catalytic subunit of the NuA4 complex or lack of the Eaf3p subunit from targeting module did not have a significant effect on strain viability. These results suggest that sufficient Esa1p is produced in the hemizygous Esa1 strain for cell survival and Eaf3p is not critical for survival. Also, whole-cell Western blotting showed that strains lacking NuA4 subunits Nbn1p or Eaf3p did not exhibit reduced H4 acetylation attributable to NuA4. However, Wang et al. [16] reported that deletion of the NuA4 catalytic subunit Esa1p resulted in a significant decrease in the acetylation level of *C. albicans* nucleosomal H4. Importantly, deletion of either the Nbn1p or Eaf3p subunits of the NuA4 complex eliminated the characteristic increase in H4 acetylation observed on the monosomic Ch5 in DAY286-1 and compromised the ability of the deletion strains to undergo transformation to a monosomic Ch5 state. Our results support a model wherein a major pathway to generation of sorbose-resistant *C. albicans* mutants involves adaptation of a monosomic Ch5 state, with a concurrent chromosome-wide increase in H4 acetylation to facilitate a twofold upregulation of the large proportion of Ch5 genes required for normal cellular homeostasis.

We find consistency in the fact that there are epigenetic changes on both aneuploid chromosomes in Sor125(55), monosomic Ch5 and accompanying trisomic Ch4/7b. Although the details of gene regulation on Ch4/7b are less understood, and will be the subject of future study, the increase in acetylation of H3 is likely to be the outcome of a mechanism distinct than that responsible for upregulating expression of single-copy genes on Ch5. This mechanism might be involved in upregulation of a subset of genes on the trisomic Ch4/7b in conjunction with the increase in gene dosage associated with sorbose adaptation, concomitant with compensatory downregulation of many other genes to the disomic level. Given that mutants were preserved immediately after generation, we believe that the increase in H3 and H4 acetylation we detected is directly coupled with generation of aneuploid states and not with an unspecified instability due to, for example, how cells were maintained and handled (see “Methods”).

Our results suggest NuA4 as a novel drug target to reduce the viability of strains resistant to anti-fungals. We have shown that a significant fraction of sorbose-resistant mutants with a monosomic Ch5 also exhibit decreased susceptibility to the anti-candidal agent caspofungin [3]. In addition, approximately a quarter of mutants that adapted to caspofungin upon direct exposure to this drug transitioned to a monosomic Ch5 state, which defines the decreased drug susceptibility [4]. Interestingly of over 20 HATs in *S. cerevisiae*, only Esa1p, the catalytic subunit of NuA4, is essential for viability [23]. In *C. albicans* NuA4, four subunits (Esa1p, Nbn1p, Eaf6p, and Epl1p) comprise the catalytic core, while eight, including Eaf3p, make up a structural/targeting module [24]. Given its role in resistance, drugs that act upon the NuA4 complex in *Candida*, but not in humans could provide a pathway to combating *C. albicans* infections.

## Conclusions

Our data imply that acetylation associated with the monosomic Ch5 requires critical subunits of the *C. albicans* NuA4 HAT complex, suggesting a mechanism similar to that operative on the single X chromosome in *Drosophila* males, and setting a precedent for a fundamental cellular process appearing earlier in evolution. Recently, transcriptional dosage compensation was reported for sets of genes on different trisomic or higher ploidy aneuploid chromosomes in wild or laboratory strains of *S. cerevisiae* [25]. However, to our knowledge, no report is available regarding the state of histone acetylation on aneuploid chromosomes in *S. cerevisiae*. It will be interesting in future experiments to determine how NuA4 HAT activity contributes to the generation of monosomic Ch5 strains and the role of increase in H3 acetylation on Ch4/7b.

## Methods

### Strains, media, and growth conditions

The parental strain BWP17 [26] and a derivative strain Esa1 in which one copy of the gene *ESA1* encoding a catalytic subunit of NuA4 HAT complex is disrupted were supplied from the Tag-Module collection, University of Toronto. The parental strain DAY286 [26], a derivative strain Nbn1, lacking the *NBN1* gene encoding a subunit from the catalytic module of the NuA4 complex, and a derivative strain Eaf3, lacking the *EAF3* (orf19.2660) gene encoding a subunit identified as a component of the targeting module of yeast *S. cerevisiae* NuA4, were supplied by Fungal Genetics Stock Center (FGSC), University of Missouri, Kansas City (http://www.fgsc.net/candida/FGSCcandidaresources.htm). All the above strains cannot utilize sorbose, Sou^−^, and die on medium in which toxic sorbose is available as the only carbon source [27]. Also, we used the Sou^−^ strain 3153A and its Sou^+^ derivative Sor125(55) [7] (see Fig. 1a for schematics of chromosome ploidy of these strains). See Table 1 for strains used in this work.

Yeast extract/pepton/dextrose (YPD), synthetic dextrose (SD), l-sorbose, or sorbitol media were previously described [11, 28]. Media were supplemented with uridine (50 µg/ml), histidine (20 µg/ml), or arginine (10 µg/ml) when needed, as previously indicated [28]. Strains were preserved as − 70 °C stocks immediately after generation of mutant colonies and handled such that to avoid accumulation of unspecified mutations and chromosome instability due to, for example, long-term propagation, the use of aged cultures or exposure to low temperatures [see 29, 30 for more].

### Generation and analysis of Ch5 monosomic mutants

Mutants that lost one Ch5 were generated by plating cells on solid synthetic medium in which l-sorbose is substituted for glucose as described [3, 18]. Intact chromosomes were prepared and precisely separated using contour-clamped homogenous electrophoretic field (CHEF) version of PFGE, as described [30].

### ChIP-Chip analysis

We performed chromatin immunoprecipitation (ChIP) as described previously [31]. Briefly, cells were transferred from − 70 °C stocks to sorbitol master plates as a streak and incubated overnight, and approximately 3500 cfu were plated on each sorbitol plate to generate independent colonies. Upon reaching approximately 1 × 10^5^ cells per colony, cells were collected and frozen at − 70 °C. Next, cells were removed from − 70 °C and proteins and DNA were formaldehyde cross-linked, cells were lysed, and whole-cell extract was prepared, and chromatin was sonicated with a Biorupter™ sonicator (Diagenode, Denville, NJ) to fragments from approximately 200 to 500 bp and then incubated with antibody specifically reactive to H4 acetylated at lysines K5, K8, K12, and K16 (pan-acetylation) (catalog #04-557, Upstate Biotechnology/Millipore, Billerica, MA), or antibody specifically reactive to H3 acetylated at K9 and K14 (catalog #39140, Active Motif, Carlsbad, CA) and immunoprecipitation performed. The cross-linking was reversed and ChIP and input control samples purified and amplified with WGA2 GenomePlex Complete Whole Genome Amplification (WGA) kit from Sigma-Aldrich Corporation (St. Louis, MO). Custom-designed tiling microarrays were generously provided by NimbleGen Inc. (Madison, WI) (http://www.nimblegen.com). Each microarray contained 710,907 probes that permitted up to four matches within the genome. Of all probes, approximately 20,000 matched the Ch5 sequences. The probes were in situ synthesized 50-mers with an average probe spacing of 35 bp on each strand tiled in an unbiased fashion across the entire genome. Empty features on the array were filled with randomly generated probes that had G + C content and length comparable to the *C. albicans* probes as controls for non-specific binding.

Array hybridizations were performed by Roche NimbleGen. Each array was hybridized with a mixture of ChIP-selected DNA fragments labeled with Cy5 and control input DNA from the same strain labeled with Cy3. We obtained the data from two independent experiments each for H4 and H3 acetylation. Signal intensities were scanned and normalized, and the ratio of ChIP DNA/Input DNA for either Sor125(55) or 3153A and difference between log2 ratios Sor125(55)/Input DNA and log2 ratios 3153A/Input DNA for either H4 or H3 acetylation were determined and are presented independently for each repeat in Additional file 6: Table S2. Raw data are available at GEO with the accession number GSE81684. An analysis was performed using an algorithm written in Python and plotted as the position of the probes on each chromosome in the reference strain SC5314, as annotated in genomic assambly 21 in CGD, versus the averaged log2 ratio Sor125(55) minus log2 ratio 3153A. Thus, the graphs reflect the difference in acetylation with data plotted above or below the *X*-axis reflecting increased or decreased acetylation in aneuploid cells compared to that in disomic parental cells, respectively. Thus, a score of +1 reflects a twofold increase in acetylation at a particular locus in the mutant compared to the parental strain. To minimize random noise on the plots, which obscured real effects due to the high probe density, log2 ratio differences were counted as significant and plotted only if the following criteria were met: mean log2 ratio difference of 0.2 units or greater (in the same direction) over three consecutive probes and *p* < 0.1 for all three consecutive probes. The use of three consecutive probes for this analysis is based on the fact that the average size of the immunoprecipitated DNA fragments was similar to the size of a genomic region covered by three probes. Thus, each dot corresponds to the location of three consecutive probes on the chromosome that satisfied a high stringency statistical filter.

### Western blot analysis

*Candida albicans* cells were seeded on YPD plates (3000 cfu/plate) and incubated at 37 °C. Cells were collected in wash buffer containing 20 mM Tris, pH 8, 0.1 M NaCl, 0.5 mM dithiothreitol, 5 mM EDTA and washed three times in this buffer. Cells were suspended in lysis buffer containing 20 mM Tris, pH 8, 0.1 M NaCl, 0.1% NP-40, 0.5 mM dithiothreitol, 5 mM EDTA, 10 mM Sodium butyrate, and Protease Inhibitor Mini Tablets (Thermo Scientific, Rockford, IL), then microglass beads (Biospec Products, Bartlesville, OK) added, and cell lysed in a BeadBeater (Mini-Beadbeater-1, Biospec Products) at 20 s duration with 10 ~ 1-min intervals on ice. The lysed cells were mixed with 0.4 N HCl to a final concentration of 0.2 N, incubated on ice for 30 min with occasional vortexing, and centrifuged at 14,000 rpm for 10 min at 4 °C. Supernatants were collected, and 1/5 volume of 100% trichloroacetic acid was added, and after 30 min of incubation on ice, the samples were centrifuged at 14,000 rpm for 10 min at 4 °C and supernatants were discarded. The pellets were resuspended with ice-cold acetone containing 0.05 N HCl and centrifuged at 14,000 rpm for 10 min at 4 °C. The pellets were rinsed with ice-cold acetone lacking HCl twice more than resuspended with 20 mM Tris, pH 8.

Histone preparations were divided into 1 µg samples and were separated on a series of 15% SDS-PAGE gels and then transferred to PVDF membrane (0.2-µm pore size, Thermo Scientific) for Western blots; thus, H4 pan-acetylation and total H4 blots were determined for same histone preparation with the same amount of histone H4. Each Western blot analysis was conducted from three independent cell cultures. The membranes were probed with anti-histone H4, anti-pan-acetylated H4, anti-histone H4acK5, anti-histone H4acK8, anti-histone H4acK12, or anti-histone H4acK16 antibody (catalog ##39270, 39244, 39584, 39173, 39166, or 39168, respectively, Active Motif, Carlsbad, CA) followed by the incubation with ALP-labeled secondary antibody (anti-rabbit IgG linked to alkaline phosphatase, ImmunoReagents, Inc, Raleigh, NC) and detected by chemifluorescence (ECF substrate; GE Healthcare, Piscataway, NJ, USA). Fluorescence signals were detected using Molecular Imager Gel Doc XR + system (Bio-Rad, Hercules, CA), and the band densities and background corrections were quantified by Image Lab software (Bio-Rad). Acetylation levels were obtained as a density value of each histone H4 acetylation divided by density of H4. Then, acetylation levels of mutant versus parent were calculated. Data were subjected to statistical analysis (Student’s *t* test).

## Additional files

**Additional file 1: Table S1.** Positive and negative peaks of H3 or H4 acetylation presented for individual chromosomes of *Candida albicans.*

**Additional file 2: Figure S1.** Chromosome separation with PFGE of *C. albicans* mutants that adapted to utilize toxic l-sorbose. Names of the mutants and their parental strains are indicated on a top. Top gel shows precise separation of three smallest chromosomes 7, 6, and 5, as indicated on the right, while longer chromosomes are compressed in a top portion of the gel. Note that each of these chromosomes is presented by two bands, because homologous chromosomes in each pair are not of the equal size. Bottom gel shows precise separation of chromosomes 4, 3, 2, 1, and R, as indicated on the right. Note the lack of one chromosome 5 in the mutants. Also shown are chromosomes of the *Saccharomyces cerevisiae* strain 867 that serve as markers of *C. albicans* chromosomes.

**Additional file 3: Figure S2.** Example of Western blot with histone H4 antibodies of *C. albicans* strains as indicated on the top. Antibodies are indicated on the left. Purified histone extract from each strain was prepared and subjected to electrophoresis on 15% polyacrylamide gels followed by Western blot analysis (Methods).

**Additional file 4: Figure S3.** Histone H3 acetylation. (A) Example of Western blot with histone H3 antibodies of *C. albicans* strains as indicated on the top. For details, see the legend of Fig. S2. (B) Relative amount of H3 acetylation calculated from three independent Western blot analyses.

**Additional file 5: Figure S4.** The survival of DAY286, Nbn1 (*nbn1* −/−), and Eaf3 (*eaf3* −/−) Sou^−^ cells on l-sorbose medium. Daily survival rate was measured according to (18).

**Additional file 6: Table S2.** ChIP data (http://www.ncbi.nlm.nih.gov/geo/query/acc.cgi?acc=GSE81684).

## Abbreviations

ChIP: chromatin immunoprecipitation
HAT: histone acetyltransferase
ORF: open reading frame
CHEF: contour-clamped homogenous electrophoretic field
PFGE: pulse-field gel electrophoresis
PVDF: polyvinylidene difluoride
