# Correction to: NuA4 histone acetyltransferase activity is required for H4 acetylation on a dosage-compensated monosomic chromosome that confers resistance to fungal toxins

**DOI:** 10.1186/s13072-017-0161-1

**Published:** 2017-11-07

**Authors:** Hironao Wakabayashi, Christopher Tucker, Gabor Bethlendy, Anatoliy Kravets, Stephen L. Welle, Michael Bulger, Jeffrey J. Hayes, Elena Rustchenko

**Affiliations:** 1 Department of Biochemistry and Biophysics, University of Rochester Medical Center, Rochester, NY USA; 2 Roche Diagnostics Corporation, Indianapolis, IN USA; 3 Present Address: Parabase Genomics, Dorchester, MA USA; 4 Department of Medicine, University of Rochester Medical Center, Rochester, NY USA; 5 Department of Pediatrics, Center for Pediatric Biochemical Research, University of Rochester Medical Center, Rochester, NY USA

## Correction to: Epigenetics & Chromatin (2017) 10:49 10.1186/s13072-017-0156-y

After the publication of this work [1], it was noticed that an initial was missing from the author name: Jeffrey Hayes. His name should be written as: Jeffrey J. Hayes.

The original article has been corrected.
