# Supplementary material for: NuA4 histone acetyltransferase activity is required for H4 acetylation on a dosage-compensated monosomic chromosome that confers resistance to fungal toxins
Source: Epigenetics Chromatin. 2017 Oct 23;10:49. doi: 10.1186/s13072-017-0156-y (PMC5653997; doi:10.1186/s13072-017-0156-y)
Supplement: Supplementary file 1 — Additional file 1: Table S1. Positive and negative peaks of H3 or H4 acetylation presented for individual chromosomes of Candida albicans. [file 13072_2017_156_MOESM1_ESM.docx]

Table S1. Positive and negative peaks of H3 or H4 acetylation presented for individual chromosomes of *Candida albicans*.

Ch1 - H3 acetylation

| Contig | Probe sequence start | Probe sequence end | Log2 value of Sor125(55) mutant 1 | Log2 value of Sor125(55) mutant 2 | Log2 value of 3153A parent 1 | Log2 value of 3153A parent 2 | Probe Name | Mean difference | p value |
| --- | --- | --- | --- | --- | --- | --- | --- | --- | --- |
| 10020 | 14409 | 14465 | 0.16 | 0.26 | -0.19 | -0.02 | CONTIG19_10020FS000014409 | 0.315 | 0.070447 |
| 10052 | 32715 | 32771 | -0.26 | 0.34 | 0.02 | 0.55 | CONTIG19_10052FS000032715 | -0.245 | 0.090334 |
| 10052 | 32747 | 32804 | -0.17 | 0.05 | 0.09 | 0.4 | CONTIG19_10052RS000032747 | -0.305 | 0.093255 |
| 10052 | 57457 | 57506 | -0.02 | 0.31 | -0.27 | 0.01 | CONTIG19_10052FS000057457 | 0.275 | 0.057716 |
| 10052 | 97421 | 97470 | 0.06 | 0.38 | -0.26 | -0.05 | CONTIG19_10052RS000097421 | 0.375 | 0.09271 |
| 10097 | 47694 | 47750 | 0.19 | 0.1 | 0.71 | 0.67 | CONTIG19_10097FS000047694 | -0.545 | 0.029182 |
| 10159 | 26685 | 26745 | 0.11 | 0.26 | 0.42 | 0.49 | CONTIG19_10159RS000026685 | -0.27 | 0.093633 |
| 10159 | 41860 | 41909 | 0.03 | 0.15 | 0.27 | 0.38 | CONTIG19_10159FS000041860 | -0.235 | 0.013543 |
| 10163 | 11424 | 11482 | 0.23 | 0.17 | -0.12 | -0.12 | CONTIG19_10163RS000011424 | 0.32 | 0.059509 |
| 10163 | 42822 | 42885 | 0.25 | 0.34 | -0.14 | -0.09 | CONTIG19_10163RS000042822 | 0.41 | 0.03103 |
| 10163 | 117872 | 117927 | -0.04 | -0.03 | 0.34 | 0.36 | CONTIG19_10163RS000117872 | -0.385 | 0.008267 |
| 10173 | 296 | 358 | -0.31 | 0.19 | -0.68 | -0.22 | CONTIG19_10173RS000000296 | 0.39 | 0.032619 |
| 10195 | 3359 | 3411 | 0.32 | 0.4 | -0.26 | -0.25 | CONTIG19_10195RS000003359 | 0.615 | 0.036191 |
| 10205 | 27572 | 27632 | -0.07 | 0.07 | -0.44 | -0.36 | CONTIG19_10205FS000027572 | 0.4 | 0.047657 |
| 10206 | 15137 | 15198 | -0.08 | 0.23 | -0.42 | -0.08 | CONTIG19_10206RS000015137 | 0.325 | 0.029362 |
| 10209 | 726 | 775 | -0.05 | 0.42 | 0.44 | 0.82 | CONTIG19_10209FS000000726 | -0.445 | 0.064159 |
| 10209 | 35548 | 35597 | -0.07 | -0.09 | 0.38 | 0.33 | CONTIG19_10209FS000035548 | -0.435 | 0.021944 |
| 10215 | 303754 | 303810 | 0.21 | 0.25 | -0.52 | -0.45 | CONTIG19_10215RS000303754 | 0.715 | 0.013354 |
| 10215 | 303785 | 303834 | 0.15 | 0.34 | -0.39 | -0.31 | CONTIG19_10215FS000303785 | 0.595 | 0.05868 |
| 10216 | 27342 | 27402 | 0.28 | 0.37 | -0.03 | -0.01 | CONTIG19_10216RS000027342 | 0.345 | 0.064364 |
| 10218 | 67429 | 67484 | -0.3 | 0.05 | -0.76 | -0.39 | CONTIG19_10218FS000067429 | 0.45 | 0.014145 |
| 10218 | 67455 | 67512 | -0.18 | 0.15 | -0.58 | -0.22 | CONTIG19_10218RS000067455 | 0.385 | 0.024791 |
| 10223 | 22779 | 22835 | 0.09 | -0.25 | 0.56 | 0.34 | CONTIG19_10223FS000022779 | -0.53 | 0.071765 |
| 10237 | 105045 | 105104 | -0.44 | 0.15 | -0.02 | 0.53 | CONTIG19_10237FS000105045 | -0.4 | 0.031805 |
| 10237 | 105363 | 105423 | 0.03 | 0.22 | 0.35 | 0.55 | CONTIG19_10237RS000105363 | -0.325 | 0.009793 |
| 10237 | 108680 | 108738 | 0.25 | 0.33 | -0.12 | -0.02 | CONTIG19_10237RS000108680 | 0.36 | 0.017679 |
| 10237 | 148945 | 149001 | -0.23 | 0.21 | -0.52 | -0.18 | CONTIG19_10237RS000148945 | 0.34 | 0.092954 |
| 10237 | 183947 | 183998 | -0.39 | -0.08 | 0 | 0.28 | CONTIG19_10237RS000183947 | -0.375 | 0.025451 |
| 10241 | 148 | 208 | 0.11 | 0.32 | -0.27 | -0.2 | CONTIG19_10241FS000000148 | 0.45 | 0.098242 |
| 10241 | 187 | 246 | 0.19 | 0.27 | -0.13 | -0.11 | CONTIG19_10241RS000000187 | 0.35 | 0.054434 |
| 10241 | 167679 | 167734 | 0.57 | 0.56 | 0.11 | -0.03 | CONTIG19_10241RS000167679 | 0.525 | 0.078421 |
| 10241 | 167724 | 167786 | 0.21 | 0.37 | -0.17 | -0.12 | CONTIG19_10241FS000167724 | 0.435 | 0.080067 |
| 10241 | 172679 | 172739 | 0.08 | -0.41 | 0.41 | 0.04 | CONTIG19_10241FS000172679 | -0.39 | 0.09718 |
| 20051 | 39625 | 39681 | 0.1 | 0.32 | -0.25 | -0.05 | CONTIG19_20051FS000039625 | 0.36 | 0.017679 |
| 2507 | 96515 | 96565 | -0.05 | 0.25 | 0.19 | 0.44 | CONTIG19_2507FS000096515 | -0.215 | 0.073695 |

Ch2 - H3 acetylation

| Contig | Probe sequence start | Probe sequence end | Log2 value of Sor125(55) mutant 1 | Log2 value of Sor125(55) mutant 2 | Log2 value of 3153A parent 1 | Log2 value of 3153A parent 2 | Probe Name | Mean difference | p value |
| --- | --- | --- | --- | --- | --- | --- | --- | --- | --- |
| 10136 | 11856 | 11905 | 0.11 | 0.26 | -0.14 | 0.01 | CONTIG19_10136FS000011855 | 0.25 | 0 |
| 10139 | 59923 | 59987 | -0.03 | 0.03 | -0.36 | -0.38 | CONTIG19_10139FS000040374 | 0.37 | 0.068557501 |
| 10139 | 59960 | 60021 | -0.01 | 0.2 | -0.52 | -0.45 | CONTIG19_10139RS000040411 | 0.58 | 0.076463596 |
| 10139 | 148212 | 148268 | -0.16 | 0.08 | -0.48 | -0.28 | CONTIG19_10139RS000128663 | 0.34 | 0.037405118 |
| 10139 | 158827 | 158881 | -0.03 | 0.07 | 0.36 | 0.41 | CONTIG19_10139RS000139278 | -0.365 | 0.043536098 |
| 10139 | 172661 | 172716 | -0.07 | -0.03 | 0.31 | 0.4 | CONTIG19_10139FS000153112 | -0.405 | 0.039247718 |
| 10119 | 268207 | 268263 | -0.02 | 0.45 | -0.48 | -0.04 | CONTIG19_10119FS000031234 | 0.475 | 0.020097104 |
| 10119 | 431392 | 431449 | 0.1 | -0.16 | 0.31 | 0.1 | CONTIG19_10119RS000194419 | -0.235 | 0.067471738 |
| 10234 | 621046 | 621117 | 0 | 0.25 | 0.49 | 0.62 | CONTIG19_10234RS000086093 | -0.43 | 0.088260798 |
| 10234 | 621079 | 621143 | -0.05 | 0.32 | 0.46 | 0.82 | CONTIG19_10234FS000086126 | -0.505 | 0.00630296 |
| 10234 | 621120 | 621181 | 0.05 | 0.25 | 0.43 | 0.6 | CONTIG19_10234RS000086167 | -0.365 | 0.026147743 |
| 10076 | 748863 | 748921 | 0.07 | 0.09 | 0.54 | 0.66 | CONTIG19_10076RS000040615 | -0.52 | 0.061025828 |
| 10076 | 748896 | 748957 | -0.13 | 0.26 | 0.46 | 0.71 | CONTIG19_10076FS000040648 | -0.52 | 0.085186714 |
| 10076 | 748933 | 748998 | -0.1 | -0.16 | 0.54 | 0.6 | CONTIG19_10076RS000040685 | -0.7 | 0.054434361 |
| 10076 | 748973 | 749040 | -0.03 | 0.1 | 0.4 | 0.66 | CONTIG19_10076FS000040725 | -0.495 | 0.083120958 |
| 10076 | 749185 | 749243 | 0.16 | 0.38 | 0.5 | 0.71 | CONTIG19_10076FS000040937 | -0.335 | 0.009501082 |
| 10076 | 749222 | 749278 | 0.01 | 0.32 | 0.45 | 0.77 | CONTIG19_10076RS000040974 | -0.445 | 0.00715273 |
| 10076 | 749255 | 749314 | 0.04 | 0.5 | 0.64 | 1.09 | CONTIG19_10076FS000041007 | -0.595 | 0.00534962 |
| 10076 | 750115 | 750170 | -0.31 | 0.14 | 0.09 | 0.59 | CONTIG19_10076FS000041867 | -0.425 | 0.037405118 |
| 10076 | 765557 | 765616 | 0.75 | 0.54 | 0.23 | 0.05 | CONTIG19_10076FS000057309 | 0.505 | 0.01890394 |
| 10183 | 1104854 | 1104910 | -0.32 | 0.11 | -0.6 | -0.27 | CONTIG19_10183FS000081551 | 0.33 | 0.095729424 |
| 10257 | 1158338 | 1158387 | 0.03 | 0.18 | -0.34 | -0.24 | CONTIG19_10257FS000031079 | 0.395 | 0.040238719 |
| 10257 | 1158387 | 1158436 | -0.2 | -0.11 | -0.57 | -0.43 | CONTIG19_10257RS000031128 | 0.345 | 0.046051375 |
| 10257 | 1158423 | 1158472 | 0.02 | 0.28 | -0.74 | -0.34 | CONTIG19_10257FS000031164 | 0.69 | 0.064364406 |
| 10135 | 1522489 | 1522545 | 0.09 | 0.03 | 0.4 | 0.43 | CONTIG19_10135FS000031047 | -0.355 | 0.080270174 |
| 10135 | 1558846 | 1558909 | -0.05 | 0.19 | -0.39 | -0.27 | CONTIG19_10135FS000067404 | 0.4 | 0.094786285 |
| 10135 | 1558874 | 1558935 | -0.03 | 0.28 | -0.37 | -0.18 | CONTIG19_10135RS000067432 | 0.4 | 0.094786285 |
| 10135 | 1558919 | 1558981 | -0.01 | 0.37 | -0.63 | -0.3 | CONTIG19_10135FS000067477 | 0.645 | 0.024662839 |
| 10141 | 1631721 | 1631786 | 0.12 | 0.27 | -0.11 | 0 | CONTIG19_10141RS000074175 | 0.25 | 0.050821347 |
| 10141 | 1631757 | 1631816 | 0.13 | 0.32 | -0.11 | 0.14 | CONTIG19_10141FS000074211 | 0.21 | 0.090334471 |
| 10045 | 1816588 | 1816650 | 0.01 | 0.32 | -0.42 | -0.17 | CONTIG19_10045FS000021329 | 0.46 | 0.041459967 |
| 10045 | 1834717 | 1834772 | 0.19 | -0.04 | 0.5 | 0.33 | CONTIG19_10045FS000039458 | -0.34 | 0.056027234 |
| 10196 | 1858938 | 1858994 | 0.04 | 0 | -0.25 | -0.29 | CONTIG19_10196FS000005429 | 0.29 | 0 |
| 10113 | 1986401 | 1986452 | 0.07 | 0.2 | -0.25 | -0.22 | CONTIG19_10113FS000046965 | 0.37 | 0.085511686 |

Ch3 - H3 acetylation

| Contig | Probe sequence start | Probe sequence end | Log2 value of Sor125(55) mutant 1 | Log2 value of Sor125(55) mutant 2 | Log2 value of 3153A parent 1 | Log2 value of 3153A parent 2 | Probe Name | Mean difference | p value |
| --- | --- | --- | --- | --- | --- | --- | --- | --- | --- |
| 20227 | 73232 | 73290 | -0.34 | -0.12 | 0 | 0.29 | CONTIG19_20227FS000073231 | -0.375 | 0.05924621 |
| 20227 | 79760 | 79809 | -0.06 | 0.06 | 0.19 | 0.39 | CONTIG19_20227RS000079759 | -0.29 | 0.087259037 |
| 20227 | 79974 | 80031 | -0.12 | 0.38 | 0.28 | 0.73 | CONTIG19_20227RS000079973 | -0.375 | 0.042378609 |
| 20227 | 80018 | 80079 | -0.18 | -0.22 | 0.66 | 0.62 | CONTIG19_20227FS000080017 | -0.84 | 5.94971E-17 |
| 20227 | 125341 | 125400 | 0.23 | 0.22 | -0.07 | -0.13 | CONTIG19_20227RS000125340 | 0.325 | 0.048874504 |
| 20227 | 125384 | 125447 | 0.34 | 0.41 | -0.18 | -0.13 | CONTIG19_20227FS000125383 | 0.53 | 0.012010269 |
| 20227 | 125418 | 125480 | 0.34 | 0.5 | -0.03 | 0.01 | CONTIG19_20227RS000125417 | 0.43 | 0.088260798 |
| 10123 | 522503 | 522560 | -0.16 | 0.06 | 0.15 | 0.31 | CONTIG19_10123FS000266929 | -0.28 | 0.06795004 |
| 10046 | 567042 | 567100 | 0.01 | 0.08 | 0.36 | 0.52 | CONTIG19_10046FS000040664 | -0.395 | 0.072214959 |
| 10046 | 761891 | 761940 | -0.2 | -0.12 | 0.19 | 0.32 | CONTIG19_10046RS000235513 | -0.415 | 0.038304298 |
| 10259 | 809780 | 809837 | -0.11 | 0.13 | -0.5 | -0.28 | CONTIG19_10259RS000044350 | 0.4 | 0.01591218 |
| 10236 | 886430 | 886482 | -0.25 | -0.57 | 0.11 | -0.09 | CONTIG19_10236RS000030771 | -0.42 | 0.090334471 |
| 10236 | 1032306 | 1032362 | -0.29 | -0.07 | -0.55 | -0.36 | CONTIG19_10236RS000176647 | 0.275 | 0.034690338 |
| 10236 | 1068193 | 1068245 | 0.17 | 0.29 | -0.26 | -0.13 | CONTIG19_10236RS000212534 | 0.425 | 0.007489299 |
| 10236 | 1099915 | 1099968 | -0.14 | -0.14 | 0.24 | 0.28 | CONTIG19_10236RS000244256 | -0.4 | 0.031804503 |
| 10236 | 1124986 | 1125048 | 0.1 | 0.19 | -0.38 | -0.25 | CONTIG19_10236FS000269327 | 0.46 | 0.027661699 |
| 2513 | 1350803 | 1350852 | -0.22 | 0.18 | -0.58 | -0.19 | CONTIG19_2513FS000082302 | 0.365 | 0.008720273 |
| 2513 | 1350841 | 1350897 | 0.09 | 0.4 | -0.37 | 0 | CONTIG19_2513RS000082340 | 0.43 | 0.044343479 |
| 2513 | 1350870 | 1350926 | 0.04 | 0.38 | -0.27 | 0.11 | CONTIG19_2513FS000082369 | 0.29 | 0.043835403 |
| 2513 | 1357735 | 1357787 | -0.03 | -0.03 | -0.26 | -0.24 | CONTIG19_2513RS000089234 | 0.22 | 0.028917358 |
| 2514 | 1476563 | 1476622 | 0.24 | 0.21 | 0.8 | 0.71 | CONTIG19_2514FS000025291 | -0.53 | 0.03599667 |
| 2514 | 1476705 | 1476760 | 0.16 | 0.27 | 0.52 | 0.57 | CONTIG19_2514FS000025433 | -0.33 | 0.057715877 |
| 10254 | 1533995 | 1534044 | 0.39 | 0.57 | 0.03 | 0.26 | CONTIG19_10254FS000002773 | 0.335 | 0.047421037 |
| 10254 | 1617171 | 1617229 | 0.01 | -0.13 | 0.27 | 0.09 | CONTIG19_10254RS000085949 | -0.24 | 0.052929352 |
| 10254 | 1638026 | 1638080 | -0.18 | -0.07 | 0.23 | 0.48 | CONTIG19_10254RS000106804 | -0.48 | 0.0921905 |
| 2405 | 1783561 | 1783610 | -0.1 | 0.37 | -0.43 | 0.07 | CONTIG19_2405FS000015583 | 0.315 | 0.030292344 |

Ch4 - H3 acetylation

| Contig | Probe sequence start | Probe sequence end | Log2 value of Sor125(55) mutant 1 | Log2 value of Sor125(55) mutant 2 | Log2 value of 3153A parent 1 | Log2 value of 3153A parent 2 | Probe Name | Mean difference | p value |
| --- | --- | --- | --- | --- | --- | --- | --- | --- | --- |
| 10231 | 30577 | 30639 | 0.23 | 0.28 | 0.03 | 0.06 | CONTIG19_10231FS000017088 | 0.21 | 0.030292344 |
| 10231 | 51648 | 51701 | -0.11 | 0.12 | 0.21 | 0.37 | CONTIG19_10231RS000038159 | -0.285 | 0.077791862 |
| 10231 | 51690 | 51743 | -0.07 | -0.19 | 0.2 | 0.12 | CONTIG19_10231FS000038201 | -0.29 | 0.043835403 |
| 10212 | 436590 | 436646 | -0.11 | 0 | -0.54 | -0.56 | CONTIG19_10212RS000286669 | 0.495 | 0.083120958 |
| 10158 | 556104 | 556161 | 0 | -0.09 | 0.24 | 0.14 | CONTIG19_10158FS000106421 | -0.235 | 0.013543058 |
| 10158 | 592154 | 592209 | -0.22 | -0.09 | 0.29 | 0.33 | CONTIG19_10158FS000142471 | -0.465 | 0.061417113 |
| 10158 | 679454 | 679511 | -0.02 | -0.34 | 0.46 | 0.28 | CONTIG19_10158RS000229771 | -0.55 | 0.080591051 |
| 10109 | 804523 | 804580 | -0.03 | 0.07 | -0.32 | -0.26 | CONTIG19_10109RS000029829 | 0.31 | 0.0410154 |
| 10115 | 907078 | 907141 | -0.01 | 0.33 | -0.3 | 0.02 | CONTIG19_10115RS000008169 | 0.3 | 0.021212805 |
| 10192 | 964817 | 964873 | -0.41 | -0.06 | -0.81 | -0.37 | CONTIG19_10192FS000022409 | 0.355 | 0.080270174 |
| 10192 | 1015274 | 1015336 | 0.29 | 0.28 | 0.07 | 0 | CONTIG19_10192FS000072866 | 0.25 | 0.076030816 |
| 10192 | 1015309 | 1015371 | 0.37 | 0.33 | -0.08 | -0.17 | CONTIG19_10192RS000072901 | 0.475 | 0.033475417 |
| 10192 | 1024392 | 1024460 | 0.07 | 0.38 | -0.24 | 0 | CONTIG19_10192RS000081984 | 0.345 | 0.064364406 |
| 10192 | 1032637 | 1032698 | -0.02 | 0.06 | 0.3 | 0.5 | CONTIG19_10192RS000090229 | -0.38 | 0.099695851 |
| 10192 | 1065507 | 1065569 | -0.07 | 0.2 | -0.48 | -0.2 | CONTIG19_10192RS000123099 | 0.405 | 0.007859104 |
| 10192 | 1072968 | 1073017 | 0.25 | 0.14 | 0.49 | 0.37 | CONTIG19_10192FS000130560 | -0.235 | 0.013543058 |
| 10126 | 1158626 | 1158682 | -0.33 | 0.21 | -0.53 | -0.05 | CONTIG19_10126RS000005419 | 0.23 | 0.0825712 |
| 10126 | 1158658 | 1158707 | -0.11 | 0.18 | -0.4 | -0.21 | CONTIG19_10126FS000005451 | 0.34 | 0.09295429 |
| 10104 | 1241970 | 1242034 | 0.12 | 0.31 | -0.14 | -0.02 | CONTIG19_10104FS000047004 | 0.295 | 0.07517972 |
| 10104 | 1242015 | 1242073 | 0.29 | 0.4 | -0.28 | -0.13 | CONTIG19_10104RS000047049 | 0.55 | 0.023139614 |
| 10104 | 1242627 | 1242683 | 0.2 | 0.38 | -0.48 | -0.22 | CONTIG19_10104FS000047661 | 0.64 | 0.039737049 |
| 10104 | 1242653 | 1242709 | 0.18 | 0.24 | -0.25 | -0.27 | CONTIG19_10104RS000047687 | 0.47 | 0.05405016 |
| 10104 | 1245182 | 1245238 | -0.02 | 0.03 | -0.39 | -0.31 | CONTIG19_10104RS000050216 | 0.355 | 0.026883436 |
| 10104 | 1246113 | 1246169 | -0.22 | 0.17 | -0.49 | -0.08 | CONTIG19_10104RS000051147 | 0.26 | 0.024473313 |
| 10104 | 1257095 | 1257144 | -0.14 | 0.11 | 0.14 | 0.35 | CONTIG19_10104RS000062129 | -0.26 | 0.048874504 |
| 10104 | 1257246 | 1257302 | -0.37 | -0.01 | 0.14 | 0.37 | CONTIG19_10104RS000062280 | -0.445 | 0.092336413 |
| 10104 | 1257280 | 1257329 | -0.1 | 0.24 | 0.25 | 0.61 | CONTIG19_10104FS000062314 | -0.36 | 0.017679336 |
| 10104 | 1284727 | 1284784 | -0.04 | 0.19 | -0.34 | -0.13 | CONTIG19_10104FS000089761 | 0.31 | 0.020529003 |
| 2212 | 1358284 | 1358340 | 0.27 | 0.39 | -0.16 | -0.16 | CONTIG19_2212RS000001556 | 0.49 | 0.077567305 |
| 10162 | 1384006 | 1384061 | 0.05 | 0.14 | -0.14 | -0.1 | CONTIG19_10162FS000012568 | 0.215 | 0.073694607 |
| 10162 | 1406664 | 1406720 | 0.21 | 0.43 | -0.06 | 0.08 | CONTIG19_10162RS000035226 | 0.31 | 0.081693104 |
| 10162 | 1422671 | 1422727 | -0.14 | 0.27 | 0.14 | 0.51 | CONTIG19_10162FS000051233 | -0.26 | 0.048874504 |
| 10166 | 1519776 | 1519838 | 0.2 | 0.34 | -0.46 | -0.29 | CONTIG19_10166RS000031620 | 0.645 | 0.014802443 |
| 10166 | 1531331 | 1531380 | -0.11 | 0.12 | -0.43 | -0.16 | CONTIG19_10166RS000043175 | 0.3 | 0.042378609 |
| 10166 | 1531362 | 1531418 | -0.01 | 0.11 | -0.32 | -0.23 | CONTIG19_10166FS000043206 | 0.325 | 0.029361614 |
| 10166 | 1548747 | 1548803 | -0.14 | -0.05 | 0.19 | 0.35 | CONTIG19_10166FS000060591 | -0.365 | 0.060859653 |

Ch5 - H3 acetylation

| Contig | Probe sequence start | Probe sequence end | Log2 value of Sor125(55) mutant 1 | Log2 value of Sor125(55) mutant 2 | Log2 value of 3153A parent 1 | Log2 value of 3153A parent 2 | Probe Name | Mean difference | p value |
| --- | --- | --- | --- | --- | --- | --- | --- | --- | --- |
| 2350 | 1301 | 1350 | -0.11 | -0.07 | 0.37 | 0.39 | CONTIG19_2350FS000001300 | -0.47 | 0.013543058 |
| 10080 | 51640 | 51697 | -0.19 | 0.1 | -0.43 | -0.16 | CONTIG19_10080FS000030383 | 0.25 | 0.025451223 |
| 10137 | 251586 | 251650 | -0.02 | 0.18 | -0.28 | -0.02 | CONTIG19_10137RS000054554 | 0.23 | 0.0825712 |
| 10137 | 268361 | 268410 | -0.36 | -0.26 | -0.6 | -0.52 | CONTIG19_10137RS000071329 | 0.25 | 0.025451223 |
| 10137 | 273615 | 273675 | -0.3 | -0.13 | -0.79 | -0.58 | CONTIG19_10137RS000076583 | 0.47 | 0.027073869 |
| 10137 | 273642 | 273699 | -0.33 | 0 | -0.9 | -0.44 | CONTIG19_10137FS000076610 | 0.505 | 0.081493098 |
| 10137 | 281236 | 281292 | 0.28 | 0.44 | -0.1 | 0.04 | CONTIG19_10137FS000084204 | 0.39 | 0.016320008 |
| 10170 | 386412 | 386467 | -0.42 | -0.27 | -0.05 | 0.17 | CONTIG19_10170FS000014573 | -0.405 | 0.054880173 |
| 10170 | 399452 | 399512 | 0.09 | 0.07 | 0.45 | 0.46 | CONTIG19_10170RS000027613 | -0.375 | 0.025451223 |
| 10202 | 475217 | 475279 | 0.09 | 0.24 | -0.28 | -0.06 | CONTIG19_10202RS000001839 | 0.335 | 0.066272079 |
| 10202 | 475254 | 475310 | 0.08 | 0.25 | -0.15 | -0.05 | CONTIG19_10202FS000001876 | 0.265 | 0.083598005 |
| 10202 | 496297 | 496347 | -1.49 | -2.04 | -0.5 | -1.27 | CONTIG19_10202RS000022919 | -0.88 | 0.079166848 |
| 10202 | 617239 | 617288 | 0.28 | 0.5 | -0.11 | 0.21 | CONTIG19_10202FS000143861 | 0.34 | 0.09295429 |
| 10202 | 633183 | 633239 | -0.33 | -0.08 | -0.56 | -0.32 | CONTIG19_10202FS000159805 | 0.235 | 0.013543058 |

Ch6 - H3 acetylation

| Contig | Probe sequence start | Probe sequence end | Log2 value of Sor125(55) mutant 1 | Log2 value of Sor125(55) mutant 2 | Log2 value of 3153A parent 1 | Log2 value of 3153A parent 2 | Probe Name | Mean difference | p value |
| --- | --- | --- | --- | --- | --- | --- | --- | --- | --- |
| 10185 | 132128 | 132184 | 0.15 | -0.13 | 0.46 | 0.18 | CONTIG19_10185RS000010378 | -0.31 | 8.0609E-17 |
| 10176 | 283214 | 283263 | 0.05 | -0.01 | 0.25 | 0.23 | CONTIG19_10176FS000022773 | -0.22 | 0.057715877 |
| 10176 | 373358 | 373416 | 0.36 | 0.41 | 0.63 | 0.7 | CONTIG19_10176FS000112917 | -0.28 | 0.022726761 |
| 10177 | 464745 | 464794 | -0.34 | 0.02 | -0.73 | -0.36 | CONTIG19_10177FS000020162 | 0.385 | 0.008267324 |
| 10230 | 540585 | 540637 | -0.36 | -0.55 | 0.1 | 0.01 | CONTIG19_10230FS000035957 | -0.51 | 0.062214882 |
| 10230 | 658313 | 658375 | 0.11 | 0.37 | -0.17 | 0.02 | CONTIG19_10230RS000153685 | 0.315 | 0.070446575 |
| 10233 | 857707 | 857763 | -0.14 | 0.18 | 0.15 | 0.47 | CONTIG19_10233FS000138433 | -0.29 | 8.61683E-17 |
| 20101 | 876445 | 876495 | 0.06 | 0.09 | -0.24 | -0.23 | CONTIG19_20101FS000000911 | 0.31 | 0.020529003 |
| 10140 | 1018484 | 1018540 | -0.22 | 0.1 | -0.54 | -0.19 | CONTIG19_10140RS000031272 | 0.305 | 0.031283963 |

Ch7 - H3 acetylation

| Contig | Probe sequence start | Probe sequence end | Log2 value of Sor125(55) mutant 1 | Log2 value of Sor125(55) mutant 2 | Log2 value of 3153A parent 1 | Log2 value of 3153A parent 2 | Probe Name | Mean difference | p value |
| --- | --- | --- | --- | --- | --- | --- | --- | --- | --- |
| 10262 | 19666 | 19725 | 0.06 | 0.14 | -0.35 | -0.34 | CONTIG19_10262FS000019665 | 0.445 | 0.049968351 |
| 10262 | 19704 | 19766 | 0.17 | 0.27 | -0.17 | -0.19 | CONTIG19_10262RS000019703 | 0.4 | 0.094786285 |
| 10262 | 49414 | 49464 | 0.07 | 0.19 | -0.37 | -0.21 | CONTIG19_10262FS000049413 | 0.42 | 0.030292344 |
| 10262 | 109304 | 109353 | -0.08 | 0.06 | -0.56 | -0.4 | CONTIG19_10262FS000109303 | 0.47 | 0.013543058 |
| 10262 | 109349 | 109398 | -0.13 | 0.15 | -0.44 | -0.24 | CONTIG19_10262RS000109348 | 0.35 | 0.072442242 |
| 10262 | 109376 | 109432 | -0.22 | 0.17 | -0.46 | -0.12 | CONTIG19_10262FS000109375 | 0.265 | 0.059881242 |
| 10262 | 154114 | 154171 | -0.08 | 0.3 | -0.39 | -0.12 | CONTIG19_10262FS000154113 | 0.365 | 0.095212688 |
| 10262 | 170476 | 170537 | 0.07 | 0.22 | -0.21 | -0.15 | CONTIG19_10262RS000170475 | 0.325 | 0.087590437 |
| 10262 | 217361 | 217410 | -0.04 | 0.06 | 0.37 | 0.49 | CONTIG19_10262RS000217360 | -0.42 | 0.01515475 |
| 10248 | 383061 | 383116 | 0.29 | 0.05 | 0.54 | 0.35 | CONTIG19_10248RS000094057 | -0.275 | 0.057715877 |
| 10248 | 418052 | 418114 | 0.27 | 0.45 | -0.19 | -0.18 | CONTIG19_10248RS000129048 | 0.545 | 0.098495815 |
| 10248 | 442685 | 442740 | 0.03 | 0.15 | 0.59 | 0.67 | CONTIG19_10248FS000153681 | -0.54 | 0.023567738 |
| 10219 | 656940 | 656999 | 0.07 | 0.21 | -0.12 | -0.01 | CONTIG19_10219RS000053092 | 0.205 | 0.046499068 |
| 10219 | 674961 | 675010 | 0.13 | 0.45 | 0.32 | 0.67 | CONTIG19_10219FS000071113 | -0.205 | 0.046499068 |
| 10219 | 717456 | 717511 | -0.09 | -0.18 | -0.32 | -0.36 | CONTIG19_10219RS000113608 | 0.205 | 0.077255083 |
| 10110 | 760539 | 760598 | -0.12 | 0.33 | -0.3 | 0.1 | CONTIG19_10110RS000040795 | 0.205 | 0.077255083 |
| 10253 | 787292 | 787352 | 0.05 | 0.04 | -0.4 | -0.35 | CONTIG19_10253RS000008927 | 0.42 | 0.045395742 |
| 2506 | 840390 | 840439 | -0.14 | 0.08 | -0.72 | -0.42 | CONTIG19_2506FS000002346 | 0.54 | 0.047071053 |
| 2506 | 840503 | 840562 | 0.1 | 0.25 | -0.08 | 0.01 | CONTIG19_2506RS000002459 | 0.21 | 0.090334471 |
| 2506 | 849035 | 849084 | -0.1 | 0.22 | -0.55 | -0.28 | CONTIG19_2506RS000010991 | 0.475 | 0.033475417 |
| 2506 | 886919 | 886968 | 0.1 | 0.32 | -0.14 | 0.08 | CONTIG19_2506RS000048875 | 0.24 | 5.206E-17 |
| 2506 | 919937 | 919988 | 0.1 | -0.16 | 0.52 | 0.26 | CONTIG19_2506RS000081893 | -0.42 | 0 |

ChR - H3 acetylation

| Contig | Probe sequence start | Probe sequence end | Log2 value of Sor125(55) mutant 1 | Log2 value of Sor125(55) mutant 2 | Log2 value of 3153A parent 1 | Log2 value of 3153A parent 2 | Probe Name | Mean difference | p value |
| --- | --- | --- | --- | --- | --- | --- | --- | --- | --- |
| 2516 | 37738 | 37787 | 0.18 | 0.08 | -0.12 | -0.24 | CONTIG19_2516RS000037737 | 0.31 | 0.020529003 |
| 2516 | 100616 | 100678 | 0.18 | 0.48 | -0.25 | -0.1 | CONTIG19_2516FS000100615 | 0.505 | 0.093861414 |
| 2516 | 100650 | 100706 | -0.15 | 0.24 | -0.5 | -0.23 | CONTIG19_2516RS000100649 | 0.41 | 0.092507226 |
| 2516 | 135342 | 135400 | -0.07 | 0.13 | -0.33 | -0.1 | CONTIG19_2516RS000135341 | 0.245 | 0.038928129 |
| 2516 | 137316 | 137373 | -0.44 | 0.07 | -0.65 | -0.14 | CONTIG19_2516RS000137315 | 0.21 | 0 |
| 2516 | 141214 | 141263 | -0.17 | 0.12 | -0.46 | -0.18 | CONTIG19_2516RS000141213 | 0.295 | 0.010789133 |
| 10172 | 310549 | 310608 | -0.23 | 0.14 | -0.66 | -0.18 | CONTIG19_10172RS000142632 | 0.375 | 0.092709906 |
| 10190 | 503182 | 503244 | 0.09 | 0.34 | -0.32 | -0.07 | CONTIG19_10190RS000025742 | 0.41 | 0 |
| 10190 | 503215 | 503274 | 0.16 | 0.4 | -0.24 | -0.02 | CONTIG19_10190FS000025775 | 0.41 | 0.015524234 |
| 10190 | 535643 | 535700 | 0.29 | 0.21 | -0.22 | -0.35 | CONTIG19_10190FS000058203 | 0.535 | 0.029726963 |
| 10190 | 539888 | 539950 | 0.33 | 0.27 | -0.18 | -0.15 | CONTIG19_10190FS000062448 | 0.465 | 0.061417113 |
| 10148 | 744372 | 744437 | 0.2 | 0.38 | 0.44 | 0.57 | CONTIG19_10148FS000052097 | -0.215 | 0.073694607 |
| 10053 | 959013 | 959069 | 0.01 | 0.38 | -0.24 | 0.09 | CONTIG19_10053RS000064811 | 0.27 | 0.047071053 |
| 10053 | 1006966 | 1007029 | 0.24 | 0.67 | -0.37 | 0.07 | CONTIG19_10053RS000112764 | 0.605 | 0.005261201 |
| 10244 | 1042931 | 1042987 | 0.03 | 0.27 | -0.31 | -0.07 | CONTIG19_10244RS000016839 | 0.34 | 7.35E-17 |
| 10054 | 1133843 | 1133896 | -0.05 | 0.23 | 0.53 | 0.7 | CONTIG19_10054RS000012053 | -0.525 | 0.066451106 |
| 10251 | 1237135 | 1237192 | 0.05 | 0 | 0.58 | 0.47 | CONTIG19_10251RS000041491 | -0.5 | 0.038151448 |
| 10193 | 1279352 | 1279408 | -0.1 | 0.17 | -0.48 | -0.27 | CONTIG19_10193FS000002938 | 0.41 | 0.046499068 |
| 10247 | 1804930 | 1804986 | 0.1 | 0.3 | -0.34 | -0.1 | CONTIG19_10247FS000046813 | 0.42 | 0.030292344 |
| 10247 | 1869033 | 1869082 | -0.1 | 0.09 | -0.77 | -0.49 | CONTIG19_10247RS000110916 | 0.625 | 0.045757663 |
| 10247 | 1871585 | 1871641 | 0.05 | 0.36 | -0.3 | -0.07 | CONTIG19_10247FS000113468 | 0.39 | 0.065066818 |
| 2479 | 1890656 | 1890705 | 0.39 | 0.64 | -0.03 | 0.3 | CONTIG19_2479FS000006195 | 0.38 | 0.066766733 |
| 2479 | 1891042 | 1891091 | 0.35 | 0.26 | 0.02 | 0 | CONTIG19_2479RS000006581 | 0.295 | 0.07517972 |
| 2479 | 1892886 | 1892935 | -0.7 | -0.17 | -1.72 | -1.16 | CONTIG19_2479FS000008425 | 1.005 | 0.009501082 |
| 2479 | 1892916 | 1892972 | -0.2 | 0.2 | -1.02 | -0.79 | CONTIG19_2479RS000008455 | 0.905 | 0.059618121 |
| 2479 | 1893097 | 1893148 | -0.35 | -0.19 | -0.92 | -0.9 | CONTIG19_2479FS000008636 | 0.64 | 0.069354604 |
| 2479 | 1893132 | 1893181 | -0.03 | 0.23 | -0.5 | -0.36 | CONTIG19_2479RS000008671 | 0.53 | 0.071764626 |
| 2479 | 1894031 | 1894080 | 0.65 | 0.69 | 0.01 | -0.03 | CONTIG19_2479FS000009570 | 0.68 | 0.037405118 |
| 2479 | 1894066 | 1894115 | 0.69 | 0.85 | 0.03 | 0.13 | CONTIG19_2479RS000009605 | 0.69 | 0.027661699 |
| 2479 | 1894102 | 1894151 | 0.34 | 0.66 | -0.19 | 0.06 | CONTIG19_2479FS000009641 | 0.565 | 0.039386294 |
| 2479 | 1894144 | 1894193 | 0.46 | 0.52 | -0.22 | -0.13 | CONTIG19_2479RS000009683 | 0.665 | 0.01435741 |
| 2479 | 1895310 | 1895359 | 0.2 | 0.39 | -0.34 | -0.27 | CONTIG19_2479RS000010849 | 0.6 | 0.063451035 |
| 2511 | 1953365 | 1953424 | 0.13 | 0.42 | -0.71 | -0.25 | CONTIG19_2511RS000055615 | 0.755 | 0.071371894 |
| 2511 | 1967599 | 1967654 | 0.27 | 0.33 | 0.51 | 0.56 | CONTIG19_2511RS000069849 | -0.235 | 0.013543058 |
| 2511 | 1967634 | 1967695 | 0.45 | 0.38 | 0.81 | 0.65 | CONTIG19_2511FS000069884 | -0.315 | 0.090334471 |
| 2518 | 2235278 | 2235334 | 0.2 | 0.46 | -0.06 | 0.27 | CONTIG19_2518RS000164909 | 0.225 | 0.098242384 |
| 2518 | 2236692 | 2236742 | 0.35 | 0.3 | 0.52 | 0.53 | CONTIG19_2518FS000166323 | -0.2 | 0.094786285 |
| 2518 | 2246550 | 2246613 | -0.07 | 0.31 | 0.27 | 0.66 | CONTIG19_2518RS000176181 | -0.345 | 0.009225728 |
| 2518 | 2246596 | 2246660 | -0.12 | 0.38 | 0.38 | 0.75 | CONTIG19_2518FS000176227 | -0.435 | 0.094428432 |

Ch1 – H4 acetylation

| Contig | Probe sequence start | Probe sequence end | Log2 value of Sor125(55) mutant 1 | Log2 value of Sor125(55) mutant 2 | Log2 value of 3153A parent 1 | Log2 value of 3153A parent 2 | Probe Name | Mean difference | p value |
| --- | --- | --- | --- | --- | --- | --- | --- | --- | --- |
| 10237 | 59469 | 59528 | 0.62 | 0.66 | 0.04 | 0.07 | CONTIG19_10237FS000015688 | 0.585 | 0.005441062 |
| 10237 | 130176 | 130225 | 0.87 | 0.72 | 0.59 | 0.47 | CONTIG19_10237RS000086395 | 0.265 | 0.03599667 |
| 10237 | 131640 | 131696 | 0.85 | 0.76 | 0.43 | 0.45 | CONTIG19_10237RS000087859 | 0.365 | 0.095212688 |
| 10237 | 184358 | 184421 | -0.3 | -0.19 | -0.04 | 0.11 | CONTIG19_10237RS000140577 | -0.28 | 0.045395742 |
| 10237 | 197746 | 197808 | -1.24 | -1.98 | 0.81 | 0.44 | CONTIG19_10237RS000153965 | -2.235 | 0.05257574 |
| 10237 | 197790 | 197852 | -1.23 | -1.58 | 0.55 | 0.54 | CONTIG19_10237FS000154009 | -1.95 | 0.055360218 |
| 10237 | 197818 | 197867 | -0.99 | -1.73 | 0.67 | 0.28 | CONTIG19_10237RS000154037 | -1.835 | 0.060529993 |
| 10237 | 197852 | 197908 | -0.99 | -1.5 | 0.81 | 0.44 | CONTIG19_10237FS000154071 | -1.87 | 0.023819565 |
| 10237 | 197893 | 197948 | -1.26 | -1.9 | 0.54 | 0.42 | CONTIG19_10237RS000154112 | -2.06 | 0.079927445 |
| 10237 | 198114 | 198175 | -1.63 | -1.94 | 0.53 | 0.24 | CONTIG19_10237RS000154333 | -2.17 | 0.002933711 |
| 10237 | 198146 | 198214 | -1.51 | -1.92 | 0.56 | 0.2 | CONTIG19_10237FS000154365 | -2.095 | 0.007596534 |
| 10237 | 198176 | 198245 | -1.19 | -1.7 | 0.5 | 0.16 | CONTIG19_10237RS000154395 | -1.775 | 0.030462746 |
| 10237 | 198935 | 198989 | -0.74 | -0.94 | 0.22 | 0 | CONTIG19_10237FS000155154 | -0.95 | 0.006701013 |
| 10237 | 198978 | 199035 | -1.51 | -1.68 | -0.01 | -0.16 | CONTIG19_10237RS000155197 | -1.51 | 0.004215963 |
| 10237 | 199014 | 199063 | -1.57 | -1.5 | -0.01 | -0.13 | CONTIG19_10237FS000155233 | -1.465 | 0.041224791 |
| 10237 | 199045 | 199101 | -0.76 | -1.28 | 0.16 | -0.11 | CONTIG19_10237RS000155264 | -1.045 | 0.075790582 |
| 10237 | 199077 | 199139 | -1.5 | -1.86 | -0.01 | -0.21 | CONTIG19_10237FS000155296 | -1.57 | 0.032411192 |
| 10237 | 199120 | 199180 | -1.67 | -1.92 | 0.15 | -0.19 | CONTIG19_10237RS000155339 | -1.775 | 0.0161362 |
| 10237 | 199157 | 199213 | -1.5 | -1.95 | 0.24 | -0.24 | CONTIG19_10237FS000155376 | -1.725 | 0.005535685 |
| 10237 | 199184 | 199233 | -1.46 | -1.51 | -0.07 | -0.34 | CONTIG19_10237RS000155403 | -1.28 | 0.054575424 |
| 10237 | 199230 | 199280 | -0.73 | -0.93 | 0.02 | 0.1 | CONTIG19_10237FS000155449 | -0.89 | 0.0993285 |
| 10173 | 209661 | 209716 | -0.26 | -0.25 | -0.49 | -0.43 | CONTIG19_10173FS000009960 | 0.205 | 0.077255083 |
| 10173 | 266777 | 266832 | -0.11 | -0.21 | -0.35 | -0.48 | CONTIG19_10173RS000067076 | 0.255 | 0.037405118 |
| 10173 | 293593 | 293649 | -0.55 | -0.37 | -0.2 | -0.03 | CONTIG19_10173FS000093892 | -0.345 | 0.009225728 |
| 10163 | 622359 | 622408 | -0.04 | 0.05 | -0.37 | -0.2 | CONTIG19_10163RS000125021 | 0.29 | 0.087259037 |
| 10163 | 698335 | 698387 | -0.3 | 0 | -0.58 | -0.27 | CONTIG19_10163RS000200997 | 0.275 | 0.01157363 |
| 10163 | 729849 | 729901 | 0.23 | 0.14 | -0.11 | -0.2 | CONTIG19_10163FS000232511 | 0.34 | 0 |
| 10163 | 748415 | 748471 | -0.43 | -0.69 | 0.07 | -0.3 | CONTIG19_10163FS000251077 | -0.445 | 0.078286324 |
| 10163 | 748456 | 748508 | -0.46 | -0.69 | -0.09 | -0.26 | CONTIG19_10163RS000251118 | -0.4 | 0.047657259 |
| 10096 | 775261 | 775317 | -0.34 | -0.23 | -0.12 | -0.05 | CONTIG19_10096FS000007998 | -0.2 | 0.063451035 |
| 10087 | 895716 | 895771 | -0.17 | 0.02 | -0.47 | -0.25 | CONTIG19_10087RS000041705 | 0.285 | 0.033475417 |
| 10087 | 915718 | 915781 | -0.52 | -0.32 | -0.24 | -0.05 | CONTIG19_10087RS000061707 | -0.275 | 0.01157363 |
| 10256 | 932379 | 932436 | 0.54 | 0.73 | 0.09 | 0.16 | CONTIG19_10256RS000015961 | 0.51 | 0.074553742 |
| 10052 | 1128234 | 1128293 | 0.62 | 0.7 | 0.4 | 0.4 | CONTIG19_10052RS000041896 | 0.26 | 0.097179581 |
| 10150 | 1182377 | 1182440 | 0.57 | 0.52 | 0.37 | 0.27 | CONTIG19_10150FS000013851 | 0.225 | 0.070446575 |
| 10150 | 1305989 | 1306040 | -0.26 | -0.61 | 0.04 | -0.25 | CONTIG19_10150FS000137463 | -0.33 | 0.057715877 |
| 10150 | 1306023 | 1306074 | -0.47 | -0.65 | 0.14 | -0.2 | CONTIG19_10150RS000137497 | -0.53 | 0.095373572 |
| 10150 | 1306055 | 1306107 | -0.36 | -0.54 | 0.08 | -0.22 | CONTIG19_10150FS000137529 | -0.38 | 0.099695851 |
| 10241 | 1363733 | 1363782 | 0.02 | -0.02 | -0.18 | -0.29 | CONTIG19_10241FS000024531 | 0.235 | 0.094123829 |
| 10241 | 1378826 | 1378882 | -0.13 | -0.05 | -0.4 | -0.3 | CONTIG19_10241FS000039624 | 0.26 | 0.024473313 |
| 10205 | 1551103 | 1551158 | 0.59 | 0.39 | 0.33 | 0.04 | CONTIG19_10205FS000024162 | 0.305 | 0.093254724 |
| 10218 | 1700503 | 1700562 | 0.04 | -0.13 | 0.27 | 0.18 | CONTIG19_10218RS000002357 | -0.27 | 0.093632989 |
| 10218 | 1757398 | 1757456 | -0.32 | -0.36 | 0.16 | 0.01 | CONTIG19_10218RS000059252 | -0.425 | 0.081930737 |
| 10218 | 1777652 | 1777710 | -0.13 | 0.03 | 0.14 | 0.23 | CONTIG19_10218FS000079506 | -0.235 | 0.094123829 |
| 10218 | 1801691 | 1801752 | 0.32 | 0.48 | 0.01 | 0.16 | CONTIG19_10218FS000103545 | 0.315 | 0.010104227 |
| 10218 | 1811319 | 1811376 | -0.1 | 0.14 | -0.49 | -0.17 | CONTIG19_10218RS000113173 | 0.35 | 0.072442242 |
| 10218 | 1811360 | 1811409 | -0.28 | -0.1 | -0.51 | -0.38 | CONTIG19_10218FS000113214 | 0.255 | 0.062214882 |
| 10218 | 1811411 | 1811460 | 0 | 0.21 | -0.3 | -0.15 | CONTIG19_10218RS000113265 | 0.33 | 0.057715877 |
| 10215 | 1915147 | 1915210 | -0.2 | -0.38 | 0.35 | 0.05 | CONTIG19_10215FS000028440 | -0.49 | 0.077567305 |
| 10215 | 1915176 | 1915229 | -0.33 | -0.33 | 0.02 | 0.15 | CONTIG19_10215RS000028469 | -0.415 | 0.098907956 |
| 10215 | 1915222 | 1915281 | -0.32 | -0.34 | -0.05 | -0.04 | CONTIG19_10215FS000028515 | -0.285 | 0.033475417 |
| 10215 | 1915248 | 1915308 | -0.29 | -0.09 | 0.06 | 0.22 | CONTIG19_10215RS000028541 | -0.33 | 0.038535881 |
| 10215 | 2043565 | 2043624 | -0.21 | -0.23 | -0.56 | -0.51 | CONTIG19_10215FS000156858 | 0.315 | 0.070446575 |
| 10215 | 2144580 | 2144636 | 0.35 | 0.44 | -0.01 | 0.05 | CONTIG19_10215FS000257873 | 0.375 | 0.025451223 |
| 10215 | 2144968 | 2145017 | 0.38 | 0.44 | 0.08 | 0.06 | CONTIG19_10215RS000258261 | 0.34 | 0.074553742 |
| 10215 | 2177212 | 2177267 | -0.1 | 0.09 | -0.36 | -0.1 | CONTIG19_10215RS000290505 | 0.225 | 0.098242384 |
| 10215 | 2177253 | 2177308 | -0.27 | 0 | -0.49 | -0.3 | CONTIG19_10215FS000290546 | 0.26 | 0.097179581 |
| 10215 | 2232933 | 2232989 | 0.15 | 0.1 | -0.11 | -0.09 | CONTIG19_10215FS000346226 | 0.225 | 0.098242384 |
| 10215 | 2249040 | 2249100 | 0.01 | 0.16 | -0.2 | -0.08 | CONTIG19_10215RS000362333 | 0.225 | 0.042378609 |
| 10064 | 2494743 | 2494792 | 0.4 | 0.62 | -0.16 | 0.17 | CONTIG19_10064RS000034691 | 0.505 | 0.069062621 |
| 10097 | 2550193 | 2550242 | 0.13 | 0.31 | -0.24 | -0.1 | CONTIG19_10097FS000027013 | 0.39 | 0.032618594 |
| 10223 | 2654328 | 2654377 | 0.59 | 0.75 | 0.3 | 0.36 | CONTIG19_10223RS000063070 | 0.34 | 0.09295429 |
| 10223 | 2690577 | 2690626 | 0.58 | 0.61 | 0.21 | 0.19 | CONTIG19_10223FS000099319 | 0.395 | 0.040238719 |
| 10223 | 2704780 | 2704840 | 0.09 | -0.12 | 0.28 | 0.12 | CONTIG19_10223RS000113522 | -0.215 | 0.073694607 |
| 10223 | 2704817 | 2704876 | 0.02 | -0.26 | 0.35 | 0 | CONTIG19_10223FS000113559 | -0.295 | 0.07517972 |
| 10216 | 2799489 | 2799545 | -0.43 | -0.45 | -0.15 | -0.14 | CONTIG19_10216FS000006779 | -0.295 | 0.032342643 |
| 10216 | 2799515 | 2799566 | -0.42 | -0.49 | -0.06 | -0.04 | CONTIG19_10216RS000006805 | -0.405 | 0.070446575 |
| 10216 | 2857597 | 2857646 | -0.15 | -0.26 | -0.41 | -0.53 | CONTIG19_10216FS000064887 | 0.265 | 0.012010269 |
| 10216 | 2900179 | 2900235 | -0.01 | 0.1 | -0.27 | -0.21 | CONTIG19_10216FS000107469 | 0.285 | 0.055701264 |
| 10216 | 2979004 | 2979053 | -0.28 | -0.13 | -0.47 | -0.34 | CONTIG19_10216RS000186294 | 0.2 | 0.031804503 |
| 10216 | 3065905 | 3065959 | 0.18 | 0.42 | -0.12 | 0.07 | CONTIG19_10216FS000273195 | 0.325 | 0.048874504 |
| 2507 | 3150157 | 3150214 | -0.63 | -0.33 | -0.21 | -0.01 | CONTIG19_2507FS000068999 | -0.37 | 0.085511686 |

Ch2 – H4 acetylation

| Contig | Probe sequence start | Probe sequence end | Log2 value of Sor125(55) mutant 1 | Log2 value of Sor125(55) mutant 2 | Log2 value of 3153A parent 1 | Log2 value of 3153A parent 2 | Probe Name | Mean difference | p value |
| --- | --- | --- | --- | --- | --- | --- | --- | --- | --- |
| 10139 | 71814 | 71873 | -0.19 | -0.03 | -0.44 | -0.31 | CONTIG19_10139RS000052265 | 0.265 | 0.03599667 |
| 10139 | 135327 | 135383 | 0.23 | 0.36 | -0.17 | -0.04 | CONTIG19_10139RS000115778 | 0.4 | 6.2472E-17 |
| 10139 | 164240 | 164289 | 0.36 | 0.3 | 0.02 | 0.01 | CONTIG19_10139FS000144691 | 0.315 | 0.050419695 |
| 10139 | 230432 | 230490 | 0.4 | 0.39 | 0.14 | 0.17 | CONTIG19_10139FS000210883 | 0.24 | 0.052929352 |
| 10139 | 234215 | 234272 | 0.16 | 0.28 | -0.11 | -0.08 | CONTIG19_10139FS000214666 | 0.315 | 0.090334471 |
| 10119 | 265134 | 265190 | 0.53 | 0.51 | 0.06 | 0.06 | CONTIG19_10119RS000028161 | 0.46 | 0.013837381 |
| 10119 | 265170 | 265219 | 0.37 | 0.69 | -0.19 | -0.05 | CONTIG19_10119FS000028197 | 0.65 | 0.087590437 |
| 10119 | 527615 | 527672 | 0.81 | 0.81 | 0.26 | 0.29 | CONTIG19_10119FS000290642 | 0.535 | 0.017844478 |
| 10234 | 623544 | 623593 | 0.41 | 0.28 | 0.22 | 0.02 | CONTIG19_10234FS000088591 | 0.225 | 0.098242384 |
| 10076 | 849255 | 849313 | -1.07 | -1.75 | 0.4 | 0.22 | CONTIG19_10076FS000141007 | -1.72 | 0.091888462 |
| 10076 | 849290 | 849350 | -1.22 | -1.65 | 0.29 | 0.19 | CONTIG19_10076RS000141042 | -1.675 | 0.062510125 |
| 10076 | 849323 | 849385 | -0.89 | -1.44 | 0.32 | 0.19 | CONTIG19_10076FS000141075 | -1.42 | 0.093470503 |
| 10076 | 849356 | 849409 | -1.6 | -1.63 | 0.03 | 0.07 | CONTIG19_10076RS000141108 | -1.665 | 0.013380427 |
| 10076 | 850651 | 850706 | -1.61 | -2.12 | 0.15 | -0.08 | CONTIG19_10076RS000142403 | -1.9 | 0.046824206 |
| 10076 | 850693 | 850745 | -1.7 | -2.05 | 0.03 | -0.02 | CONTIG19_10076FS000142445 | -1.88 | 0.050686755 |
| 10076 | 850730 | 850783 | -1.77 | -1.85 | -0.03 | -0.2 | CONTIG19_10076RS000142482 | -1.695 | 0.016897441 |
| 10076 | 850769 | 850826 | -1.62 | -2.02 | 0.2 | -0.06 | CONTIG19_10076FS000142521 | -1.89 | 0.023567738 |
| 10076 | 907966 | 908016 | 0.11 | 0.22 | -0.24 | -0.25 | CONTIG19_10076RS000199718 | 0.41 | 0.092507226 |
| 10076 | 908007 | 908056 | -0.08 | -0.02 | -0.42 | -0.38 | CONTIG19_10076FS000199759 | 0.35 | 0.018184189 |
| 10076 | 908044 | 908093 | 0.1 | 0.18 | -0.24 | -0.13 | CONTIG19_10076RS000199796 | 0.325 | 0.029361614 |
| 10076 | 931029 | 931085 | 0.67 | 0.59 | 0.26 | 0.11 | CONTIG19_10076FS000222781 | 0.445 | 0.049968351 |
| 10076 | 931211 | 931260 | 0.83 | 0.8 | 0.26 | 0.04 | CONTIG19_10076RS000222963 | 0.665 | 0.090334471 |
| 10076 | 931249 | 931298 | 0.64 | 0.63 | 0.23 | 0.26 | CONTIG19_10076FS000223001 | 0.39 | 0.032618594 |
| 10076 | 931289 | 931338 | 0.64 | 0.74 | 0.2 | 0.22 | CONTIG19_10076RS000223041 | 0.48 | 0.052929352 |
| 10208 | 953774 | 953836 | 0.45 | 0.45 | 0.16 | 0.06 | CONTIG19_10208RS000048041 | 0.34 | 0.09295429 |
| 10183 | 1042046 | 1042114 | -0.18 | 0 | 0.08 | 0.21 | CONTIG19_10183FS000018743 | -0.235 | 0.067471738 |
| 10183 | 1085910 | 1085966 | 0.11 | 0.17 | -0.12 | -0.02 | CONTIG19_10183FS000062607 | 0.21 | 0.060448134 |
| 10257 | 1144055 | 1144110 | -0.77 | -1.24 | -0.35 | -0.7 | CONTIG19_10257RS000016796 | -0.48 | 0.079166848 |
| 10257 | 1154610 | 1154674 | 0.13 | 0.4 | -0.25 | 0.02 | CONTIG19_10257RS000027351 | 0.38 | 0 |
| 10221 | 1209814 | 1209870 | 0.17 | 0.27 | -0.05 | 0 | CONTIG19_10221FS000021021 | 0.245 | 0.064737134 |
| 10143 | 1391342 | 1391407 | 0.27 | 0.28 | -0.13 | -0.13 | CONTIG19_10143FS000003121 | 0.405 | 0.007859104 |
| 10143 | 1469373 | 1469428 | 0.14 | -0.04 | -0.12 | -0.28 | CONTIG19_10143RS000081152 | 0.25 | 0.025451223 |
| 10143 | 1473111 | 1473160 | 0.68 | 0.64 | 0.45 | 0.35 | CONTIG19_10143RS000084890 | 0.26 | 0.073132718 |
| 10135 | 1526082 | 1526143 | 0.35 | 0.43 | -0.02 | 0.01 | CONTIG19_10135FS000034640 | 0.395 | 0.040238719 |
| 10135 | 1526125 | 1526182 | 0.36 | 0.4 | 0.06 | 0.01 | CONTIG19_10135RS000034683 | 0.345 | 0.0825712 |
| 10141 | 1571205 | 1571261 | -0.9 | -0.8 | -0.41 | -0.39 | CONTIG19_10141FS000013659 | -0.45 | 0.056440087 |
| 10141 | 1614616 | 1614678 | 0.38 | 0.33 | 0.07 | 0.09 | CONTIG19_10141FS000057070 | 0.275 | 0.080591051 |
| 10184 | 1717811 | 1717860 | 0.34 | 0.28 | 0.04 | 0 | CONTIG19_10184FS000020514 | 0.29 | 0.021943711 |
| 10045 | 1842878 | 1842929 | -0.49 | -0.59 | -0.28 | -0.4 | CONTIG19_10045FS000047619 | -0.2 | 0.031804503 |
| 10125 | 2044155 | 2044214 | 0.16 | -0.12 | 0.5 | 0.16 | CONTIG19_10125RS000004621 | -0.31 | 0.061417113 |

Ch3 – H4 acetylation

| Contig | Probe sequence start | Probe sequence end | Log2 value of Sor125(55) mutant 1 | Log2 value of Sor125(55) mutant 2 | Log2 value of 3153A parent 1 | Log2 value of 3153A parent 2 | Probe Name | Mean difference | p value |
| --- | --- | --- | --- | --- | --- | --- | --- | --- | --- |
| 20227 | 70972 | 71021 | 0.48 | 0.45 | 0.09 | 0.14 | CONTIG19_20227RS000070971 | 0.35 | 0.072442242 |
| 20227 | 77877 | 77933 | 0.66 | 0.67 | 0.26 | 0.25 | CONTIG19_20227RS000077876 | 0.41 | 0.015524234 |
| 20227 | 77913 | 77962 | 0.5 | 0.58 | 0.12 | 0.17 | CONTIG19_20227FS000077912 | 0.395 | 0.024163823 |
| 20227 | 77950 | 77999 | 0.59 | 0.62 | 0.23 | 0.17 | CONTIG19_20227RS000077949 | 0.405 | 0.070446575 |
| 20227 | 109500 | 109553 | -0.8 | -0.59 | -0.54 | -0.34 | CONTIG19_20227RS000109499 | -0.255 | 0.012481141 |
| 20227 | 140224 | 140281 | -1.8 | -2.12 | -0.06 | -0.17 | CONTIG19_20227RS000140223 | -1.845 | 0.036191355 |
| 20227 | 140256 | 140309 | -1.58 | -2.07 | -0.22 | -0.37 | CONTIG19_20227FS000140255 | -1.53 | 0.070446575 |
| 20227 | 140301 | 140359 | -1.52 | -1.79 | 0.02 | 0.07 | CONTIG19_20227RS000140300 | -1.7 | 0.059741171 |
| 10169 | 248111 | 248163 | -1.84 | -1.83 | -0.13 | -0.1 | CONTIG19_10169RS000013035 | -1.72 | 0.003701236 |
| 10123 | 404981 | 405040 | 0.24 | 0.26 | -0.02 | -0.02 | CONTIG19_10123RS000149407 | 0.27 | 0.023567738 |
| 10123 | 489141 | 489197 | 0.22 | 0.13 | 0.02 | -0.08 | CONTIG19_10123RS000233567 | 0.205 | 0.015524234 |
| 10123 | 506477 | 506533 | -0.44 | -0.58 | -0.09 | -0.13 | CONTIG19_10123FS000250903 | -0.4 | 0.079166848 |
| 10123 | 506512 | 506568 | -0.21 | -0.16 | 0.02 | 0.04 | CONTIG19_10123RS000250938 | -0.215 | 0.044343479 |
| 10123 | 508167 | 508219 | -0.29 | -0.05 | -0.06 | 0.18 | CONTIG19_10123RS000252593 | -0.23 | 0 |
| 10046 | 606318 | 606375 | 0.24 | 0.16 | -0.03 | -0.09 | CONTIG19_10046FS000079940 | 0.26 | 0.024473313 |
| 10046 | 656051 | 656102 | -0.43 | -0.41 | -0.12 | -0.12 | CONTIG19_10046FS000129673 | -0.3 | 0.021212805 |
| 10236 | 924900 | 924956 | 0.35 | 0.47 | 0.04 | 0.09 | CONTIG19_10236RS000069241 | 0.345 | 0.064364406 |
| 10236 | 1031518 | 1031576 | -0.04 | -0.09 | -0.34 | -0.33 | CONTIG19_10236RS000175859 | 0.27 | 0.070446575 |
| 10236 | 1092334 | 1092396 | 0.44 | 0.35 | 0 | -0.03 | CONTIG19_10236RS000236675 | 0.41 | 0.046499068 |
| 2513 | 1332632 | 1332691 | -0.09 | -0.1 | 0.23 | 0.18 | CONTIG19_2513RS000064131 | -0.3 | 0.042378609 |
| 10254 | 1583226 | 1583284 | -0.9 | -0.85 | -0.33 | -0.36 | CONTIG19_10254FS000052004 | -0.53 | 0.047955861 |
| 10254 | 1588407 | 1588464 | 0.21 | -0.12 | 0.42 | 0.11 | CONTIG19_10254RS000057185 | -0.22 | 0.028917358 |
| 10254 | 1733327 | 1733387 | -0.11 | -0.29 | 0.07 | -0.05 | CONTIG19_10254FS000202105 | -0.21 | 0.090334471 |
| 2405 | 1785814 | 1785863 | 0.09 | 0.21 | -0.14 | 0 | CONTIG19_2405FS000017836 | 0.22 | 0.028917358 |
| 2405 | 1792004 | 1792060 | -0.77 | -0.6 | -0.4 | -0.23 | CONTIG19_2405RS000024026 | -0.37 | 0 |
| 2405 | 1792083 | 1792141 | -1.01 | -0.65 | -0.48 | -0.23 | CONTIG19_2405FS000024105 | -0.475 | 0.073387061 |
| 2405 | 1792635 | 1792692 | -1.18 | -1.02 | -0.62 | -0.49 | CONTIG19_2405FS000024657 | -0.545 | 0.017517223 |
| 2405 | 1792635 | 1792692 | -1.18 | -1.02 | -0.62 | -0.49 | CONTIG19_2405FS000024657 | -0.545 | 0.017517223 |

Ch4 – H4 acetylation

| Contig | Probe sequence start | Probe sequence end | Log2 value of Sor125(55) mutant 1 | Log2 value of Sor125(55) mutant 2 | Log2 value of 3153A parent 1 | Log2 value of 3153A parent 2 | Probe Name | Mean difference | p value |
| --- | --- | --- | --- | --- | --- | --- | --- | --- | --- |
| 10231 | 16813 | 16862 | 0.24 | 0.31 | -0.12 | -0.05 | CONTIG19_10231FS000003324 | 0.36 | 0 |
| 10231 | 19181 | 19230 | -0.2 | -0.09 | -0.45 | -0.31 | CONTIG19_10231RS000005692 | 0.235 | 0.040580253 |
| 10212 | 226427 | 226481 | -0.25 | -0.27 | -0.05 | -0.06 | CONTIG19_10212RS000076506 | -0.205 | 0.015524234 |
| 10212 | 331678 | 331742 | 0.2 | 0.33 | -0.21 | -0.07 | CONTIG19_10212FS000181757 | 0.405 | 0.007859104 |
| 10212 | 356896 | 356945 | 0.16 | 0.24 | -0.09 | -0.06 | CONTIG19_10212FS000206975 | 0.275 | 0.057715877 |
| 10158 | 462876 | 462932 | -1.87 | -2.16 | 0.05 | -0.07 | CONTIG19_10158FS000013193 | -2.005 | 0.026972717 |
| 10158 | 462919 | 462970 | -1.92 | -2.05 | -0.23 | -0.44 | CONTIG19_10158RS000013236 | -1.65 | 0.015430184 |
| 10158 | 463058 | 463107 | -1.2 | -1.68 | 0.27 | 0.21 | CONTIG19_10158RS000013375 | -1.68 | 0.079166848 |
| 10158 | 463093 | 463149 | -1.35 | -2.07 | 0.23 | 0.1 | CONTIG19_10158FS000013410 | -1.875 | 0.099347114 |
| 10158 | 463128 | 463188 | -1.28 | -1.65 | 0.24 | 0.13 | CONTIG19_10158RS000013445 | -1.65 | 0.050054521 |
| 10158 | 463275 | 463327 | -1.61 | -1.56 | 0.16 | 0.28 | CONTIG19_10158RS000013592 | -1.805 | 0.012342881 |
| 10158 | 463750 | 463811 | -1.95 | -1.91 | 0.27 | -0.02 | CONTIG19_10158FS000014067 | -2.055 | 0.051006035 |
| 10158 | 463786 | 463846 | -1.55 | -1.79 | -0.06 | 0.15 | CONTIG19_10158RS000014103 | -1.715 | 0.083047236 |
| 10158 | 463817 | 463881 | -1.82 | -1.76 | -0.05 | 0.15 | CONTIG19_10158FS000014134 | -1.84 | 0.024207556 |
| 10158 | 463852 | 463914 | -1.16 | -1.27 | 0.05 | 0.26 | CONTIG19_10158RS000014169 | -1.37 | 0.074014463 |
| 10158 | 463894 | 463951 | -1.3 | -1.63 | 0.27 | 0.11 | CONTIG19_10158FS000014211 | -1.655 | 0.03266778 |
| 10158 | 463920 | 463979 | -1.85 | -1.99 | 0.06 | 0 | CONTIG19_10158RS000014237 | -1.95 | 0.013057036 |
| 10158 | 463961 | 464018 | -1.33 | -1.67 | 0.12 | 0.11 | CONTIG19_10158FS000014278 | -1.615 | 0.064816751 |
| 10158 | 463998 | 464057 | -1.37 | -1.63 | 0.21 | 0.31 | CONTIG19_10158RS000014315 | -1.76 | 0.064883248 |
| 10158 | 464028 | 464086 | -1.66 | -1.69 | 0.32 | 0.3 | CONTIG19_10158FS000014345 | -1.985 | 0.001603573 |
| 10158 | 464071 | 464125 | -1.65 | -1.94 | 0.18 | -0.25 | CONTIG19_10158RS000014388 | -1.76 | 0.025306766 |
| 10158 | 464110 | 464166 | -1.34 | -1.39 | 0.47 | 0.18 | CONTIG19_10158FS000014427 | -1.69 | 0.04512803 |
| 10158 | 464137 | 464193 | -1.23 | -1.68 | 0.32 | 0.24 | CONTIG19_10158RS000014454 | -1.735 | 0.067626126 |
| 10158 | 464180 | 464235 | -1.15 | -1.58 | 0.32 | 0.09 | CONTIG19_10158FS000014497 | -1.57 | 0.040494328 |
| 10158 | 464211 | 464260 | -1.24 | -1.51 | 0.22 | 0.2 | CONTIG19_10158RS000014528 | -1.585 | 0.050102905 |
| 10158 | 464247 | 464308 | -1.6 | -1.58 | 0.42 | 0.11 | CONTIG19_10158FS000014564 | -1.855 | 0.056477921 |
| 10158 | 464280 | 464340 | -1.73 | -1.73 | 0.26 | 0.23 | CONTIG19_10158RS000014597 | -1.975 | 0.004834994 |
| 10158 | 464319 | 464377 | -1.66 | -2.28 | 0.16 | 0.07 | CONTIG19_10158FS000014636 | -2.085 | 0.080481788 |
| 10158 | 464611 | 464669 | -1.64 | -1.82 | 0.53 | 0.17 | CONTIG19_10158FS000014928 | -2.08 | 0.027528876 |
| 10158 | 464639 | 464702 | -1.73 | -2.19 | 0.52 | 0.2 | CONTIG19_10158RS000014956 | -2.32 | 0.019202529 |
| 10158 | 464674 | 464732 | -1.31 | -1.71 | 0.67 | 0.25 | CONTIG19_10158FS000014991 | -1.97 | 0.003231545 |
| 10158 | 464710 | 464765 | -1.71 | -1.71 | 0.37 | 0.12 | CONTIG19_10158RS000015027 | -1.955 | 0.040649256 |
| 10158 | 464859 | 464908 | -0.74 | -1.73 | 0.83 | 0.31 | CONTIG19_10158RS000015176 | -1.805 | 0.082420413 |
| 10158 | 464899 | 464948 | -0.64 | -1.14 | 0.96 | 0.5 | CONTIG19_10158FS000015216 | -1.62 | 0.007859104 |
| 10158 | 464926 | 464982 | -1.42 | -1.93 | 0.85 | 0.2 | CONTIG19_10158RS000015243 | -2.2 | 0.020249252 |
| 10158 | 464967 | 465029 | -1.51 | -1.7 | 0.77 | 0.37 | CONTIG19_10158FS000015284 | -2.175 | 0.030709526 |
| 10158 | 465003 | 465065 | -1.36 | -1.57 | 0.47 | 0.31 | CONTIG19_10158RS000015320 | -1.855 | 0.008579262 |
| 10158 | 465036 | 465097 | -1.56 | -1.9 | 0.72 | 0.34 | CONTIG19_10158FS000015353 | -2.26 | 0.005633656 |
| 10158 | 465073 | 465129 | -1.28 | -1.78 | 0.81 | 0.38 | CONTIG19_10158RS000015390 | -2.125 | 0.010484554 |
| 10158 | 465109 | 465164 | -1.26 | -1.7 | 0.5 | 0.23 | CONTIG19_10158FS000015426 | -1.845 | 0.029308642 |
| 10158 | 465146 | 465201 | -1.44 | -1.57 | 0.46 | 0.17 | CONTIG19_10158RS000015463 | -1.82 | 0.027965285 |
| 10158 | 465186 | 465235 | -1.13 | -1.38 | 0.51 | 0.24 | CONTIG19_10158FS000015503 | -1.63 | 0.003905594 |
| 10158 | 465215 | 465266 | -1.38 | -2.08 | 0.13 | -0.03 | CONTIG19_10158RS000015532 | -1.78 | 0.095835369 |
| 10158 | 465255 | 465311 | -1.41 | -1.91 | 0.43 | 0.02 | CONTIG19_10158FS000015572 | -1.885 | 0.015194933 |
| 10158 | 465287 | 465344 | -1.41 | -1.72 | 0.07 | -0.04 | CONTIG19_10158RS000015604 | -1.58 | 0.040238719 |
| 10158 | 465331 | 465388 | -1.22 | -1.51 | -0.08 | -0.07 | CONTIG19_10158FS000015648 | -1.29 | 0.073694607 |
| 10158 | 465365 | 465416 | -1.51 | -1.86 | 0 | -0.06 | CONTIG19_10158RS000015682 | -1.655 | 0.055634294 |
| 10158 | 465395 | 465451 | -1.44 | -1.7 | -0.02 | -0.26 | CONTIG19_10158FS000015712 | -1.43 | 0.004451814 |
| 10158 | 466052 | 466114 | -1.35 | -1.24 | 0.22 | 0.03 | CONTIG19_10158FS000016369 | -1.42 | 0.067000098 |
| 10158 | 466079 | 466141 | -1 | -1.57 | 0.29 | 0.12 | CONTIG19_10158RS000016396 | -1.49 | 0.084944592 |
| 10158 | 466123 | 466185 | -0.93 | -1.49 | 0.31 | 0.13 | CONTIG19_10158FS000016440 | -1.43 | 0.0840933 |
| 10158 | 466159 | 466221 | -1 | -1.41 | 0.36 | 0.36 | CONTIG19_10158RS000016476 | -1.565 | 0.082918984 |
| 10158 | 466191 | 466249 | -1.17 | -1.32 | 0.49 | 0.19 | CONTIG19_10158FS000016508 | -1.585 | 0.030101511 |
| 10158 | 466223 | 466280 | -1.44 | -1.94 | 0.25 | 0.17 | CONTIG19_10158RS000016540 | -1.9 | 0.070078799 |
| 10158 | 466268 | 466326 | -1.3 | -1.88 | 0.28 | 0.09 | CONTIG19_10158FS000016585 | -1.775 | 0.069659167 |
| 10158 | 466303 | 466365 | -1.32 | -1.99 | 0.47 | 0.27 | CONTIG19_10158RS000016620 | -2.025 | 0.07355033 |
| 10158 | 466331 | 466391 | -1.13 | -1.9 | 0.76 | 0.26 | CONTIG19_10158FS000016648 | -2.025 | 0.042378609 |
| 10158 | 466373 | 466433 | -0.86 | -1.5 | 0.62 | 0.37 | CONTIG19_10158RS000016690 | -1.675 | 0.073781815 |
| 10158 | 466412 | 466469 | -0.93 | -1.66 | 0.64 | 0.41 | CONTIG19_10158FS000016729 | -1.82 | 0.086903912 |
| 10158 | 466439 | 466500 | -1.36 | -1.93 | 0.51 | 0.31 | CONTIG19_10158RS000016756 | -2.055 | 0.057157193 |
| 10158 | 466481 | 466538 | -1.34 | -1.87 | 0.96 | 0.35 | CONTIG19_10158FS000016798 | -2.26 | 0.01126643 |
| 10158 | 466510 | 466568 | -1.63 | -1.99 | 0.68 | 0.46 | CONTIG19_10158RS000016827 | -2.38 | 0.018718715 |
| 10158 | 466547 | 466604 | -1.21 | -1.89 | 0.65 | 0.41 | CONTIG19_10158FS000016864 | -2.08 | 0.067085361 |
| 10158 | 466591 | 466644 | -1.29 | -1.8 | 0.47 | 0.3 | CONTIG19_10158RS000016908 | -1.93 | 0.055930966 |
| 10158 | 466624 | 466680 | -1.47 | -1.89 | 0.6 | 0.59 | CONTIG19_10158FS000016941 | -2.275 | 0.057211224 |
| 10158 | 466872 | 466932 | -1.59 | -1.77 | 0.64 | 0.38 | CONTIG19_10158RS000017189 | -2.19 | 0.011626466 |
| 10158 | 466906 | 466965 | -1.27 | -1.66 | 0.64 | 0.37 | CONTIG19_10158FS000017223 | -1.97 | 0.019383443 |
| 10158 | 466952 | 467002 | -1.03 | -1.02 | 0.52 | 0.37 | CONTIG19_10158RS000017269 | -1.47 | 0.034611831 |
| 10158 | 466978 | 467033 | -0.94 | -1.39 | 0.48 | 0.28 | CONTIG19_10158FS000017295 | -1.545 | 0.05139451 |
| 10158 | 467023 | 467082 | -1.36 | -1.77 | 0.4 | 0.28 | CONTIG19_10158RS000017340 | -1.905 | 0.048363368 |
| 10158 | 467058 | 467119 | -1.11 | -1.19 | 0.31 | 0.38 | CONTIG19_10158FS000017375 | -1.495 | 0.031910694 |
| 10158 | 467087 | 467145 | -1.28 | -1.61 | 0.45 | 0.54 | CONTIG19_10158RS000017404 | -1.94 | 0.068645166 |
| 10158 | 467240 | 467292 | -1.34 | -1.5 | 0.45 | 0.2 | CONTIG19_10158RS000017557 | -1.745 | 0.016413491 |
| 10158 | 467283 | 467332 | -1.47 | -1.53 | 0.22 | 0.27 | CONTIG19_10158FS000017600 | -1.745 | 0.020058739 |
| 10158 | 467313 | 467366 | -1.44 | -1.47 | 0.28 | 0.21 | CONTIG19_10158RS000017630 | -1.7 | 0.007489299 |
| 10158 | 467358 | 467411 | -1.59 | -1.45 | 0.35 | 0.05 | CONTIG19_10158FS000017675 | -1.72 | 0.080988359 |
| 10158 | 467388 | 467442 | -1.41 | -1.76 | 0.36 | 0.23 | CONTIG19_10158RS000017705 | -1.88 | 0.037206609 |
| 10158 | 467424 | 467473 | -1.22 | -1.49 | 0.48 | 0.26 | CONTIG19_10158FS000017741 | -1.725 | 0.009225728 |
| 10158 | 467461 | 467519 | -1.25 | -1.55 | 0.56 | 0.01 | CONTIG19_10158RS000017778 | -1.685 | 0.047140637 |
| 10158 | 467492 | 467548 | -1.55 | -1.54 | 0.64 | 0.06 | CONTIG19_10158FS000017809 | -1.895 | 0.098315274 |
| 10158 | 467532 | 467581 | -1.19 | -1.79 | 0.39 | 0.06 | CONTIG19_10158RS000017849 | -1.715 | 0.050009803 |
| 10158 | 467571 | 467620 | -1.24 | -1.97 | 0.57 | 0.14 | CONTIG19_10158FS000017888 | -1.96 | 0.048626115 |
| 10158 | 467603 | 467652 | -1.09 | -1.33 | 0.21 | 0.11 | CONTIG19_10158RS000017920 | -1.37 | 0.032499755 |
| 10158 | 468217 | 468266 | -1.51 | -1.8 | -0.07 | -0.16 | CONTIG19_10158FS000018534 | -1.54 | 0.04128099 |
| 10158 | 468257 | 468306 | -1.53 | -1.89 | 0.11 | -0.22 | CONTIG19_10158RS000018574 | -1.655 | 0.00576981 |
| 10158 | 468284 | 468333 | -0.92 | -1.53 | 0.22 | -0.14 | CONTIG19_10158FS000018601 | -1.265 | 0.062703536 |
| 10158 | 468320 | 468380 | -1.68 | -1.72 | -0.03 | -0.02 | CONTIG19_10158RS000018637 | -1.675 | 0.009501082 |
| 10158 | 468365 | 468422 | -1.57 | -1.52 | 0.01 | -0.04 | CONTIG19_10158FS000018682 | -1.53 | 0.020797166 |
| 10158 | 468510 | 468570 | -1.65 | -1.79 | 0.1 | -0.08 | CONTIG19_10158FS000018827 | -1.73 | 0.007359438 |
| 10158 | 468546 | 468610 | -1.79 | -1.97 | 0.01 | -0.12 | CONTIG19_10158RS000018863 | -1.825 | 0.008720273 |
| 10158 | 468762 | 468823 | -1.91 | -1.92 | 0.33 | 0.11 | CONTIG19_10158RS000019079 | -2.135 | 0.031283963 |
| 10158 | 468789 | 468849 | -1.62 | -1.93 | 0.04 | 0.18 | CONTIG19_10158FS000019106 | -1.885 | 0.075631264 |
| 10158 | 468826 | 468880 | -1.16 | -1.81 | 0.31 | -0.1 | CONTIG19_10158RS000019143 | -1.59 | 0.047955861 |
| 10158 | 468870 | 468929 | -1.63 | -1.79 | 0.09 | 0.1 | CONTIG19_10158FS000019187 | -1.805 | 0.029957193 |
| 10158 | 508395 | 508451 | 0.2 | 0.38 | -0.11 | 0.04 | CONTIG19_10158RS000058712 | 0.325 | 0.029361614 |
| 10158 | 538666 | 538720 | -0.11 | -0.07 | -0.51 | -0.48 | CONTIG19_10158FS000088983 | 0.405 | 0.007859104 |
| 10158 | 551534 | 551592 | 0.23 | 0.25 | 0.65 | 0.59 | CONTIG19_10158RS000101851 | -0.38 | 0.066766733 |
| 10115 | 917119 | 917175 | -0.06 | 0.14 | -0.4 | -0.17 | CONTIG19_10115FS000018210 | 0.325 | 0.029361614 |
| 10192 | 976078 | 976135 | 0.08 | -0.1 | 0.31 | 0.08 | CONTIG19_10192FS000033670 | -0.205 | 0.077255083 |
| 10192 | 976111 | 976164 | 0.02 | -0.41 | 0.38 | 0.04 | CONTIG19_10192RS000033703 | -0.405 | 0.070446575 |
| 20072 | 1094602 | 1094653 | 0.13 | 0.1 | -0.44 | -0.45 | CONTIG19_20072FS000004323 | 0.56 | 0.011367002 |
| 10104 | 1196840 | 1196896 | -0.24 | -0.07 | 0 | 0.12 | CONTIG19_10104RS000001874 | -0.215 | 0.073694607 |
| 10104 | 1260043 | 1260099 | 0.4 | 0.39 | -0.09 | 0.03 | CONTIG19_10104FS000065077 | 0.425 | 0.096616699 |
| 10104 | 1260085 | 1260144 | 0.18 | 0.22 | -0.03 | -0.05 | CONTIG19_10104RS000065119 | 0.24 | 0.079166848 |
| 10104 | 1322698 | 1322752 | 0.37 | 0.23 | 0.08 | -0.09 | CONTIG19_10104RS000127732 | 0.305 | 0.031283963 |
| 10166 | 1502000 | 1502056 | 0.39 | 0.49 | 0.11 | 0.13 | CONTIG19_10166RS000013844 | 0.32 | 0.079166848 |
| 10166 | 1503004 | 1503066 | 0.2 | 0.42 | -0.03 | 0.17 | CONTIG19_10166RS000014848 | 0.24 | 0.026510489 |
| 10166 | 1586656 | 1586712 | -0.59 | -0.5 | -0.32 | -0.24 | CONTIG19_10166RS000098500 | -0.265 | 0.012010269 |

Ch5 – H4 acetylation

| Contig | Probe sequence start | Probe sequence end | Log2 value of Sor125(55) mutant 1 | Log2 value of Sor125(55) mutant 2 | Log2 value of 3153A parent 1 | Log2 value of 3153A parent 2 | Probe Name | Mean difference | p value |
| --- | --- | --- | --- | --- | --- | --- | --- | --- | --- |
| 2350 | 20667 | 20723 | -1.18 | -1.18 | -0.53 | -0.54 | CONTIG19_2350RS000020666 | -0.645 | 0.004934938 |
| 2350 | 20698 | 20748 | -1.51 | -1.07 | -0.77 | -0.26 | CONTIG19_2350FS000020697 | -0.775 | 0.028731048 |
| 10080 | 39280 | 39339 | 0.03 | 0.25 | -0.61 | -0.39 | CONTIG19_10080FS000018023 | 0.64 | 0 |
| 10080 | 65994 | 66050 | -0.27 | 0.19 | -0.8 | -0.27 | CONTIG19_10080RS000044737 | 0.495 | 0.044938729 |
| 10080 | 103674 | 103736 | 0.16 | 0.16 | -0.13 | -0.18 | CONTIG19_10080FS000082417 | 0.315 | 0.050419695 |
| 10080 | 103926 | 103983 | 0.02 | 0.06 | -0.43 | -0.27 | CONTIG19_10080RS000082669 | 0.39 | 0.097179581 |
| 10080 | 108376 | 108439 | 0.34 | 0.37 | 0.02 | -0.02 | CONTIG19_10080RS000087119 | 0.355 | 0.062563142 |
| 10080 | 108414 | 108475 | 0.46 | 0.22 | -0.06 | -0.18 | CONTIG19_10080FS000087157 | 0.46 | 0.0825712 |
| 10080 | 109758 | 109820 | 0.31 | 0.29 | -0.11 | -0.04 | CONTIG19_10080FS000088501 | 0.375 | 0.076030816 |
| 10080 | 109784 | 109841 | 0.34 | 0.23 | 0.01 | -0.19 | CONTIG19_10080RS000088527 | 0.375 | 0.076030816 |
| 10080 | 109829 | 109884 | 0.26 | 0.33 | -0.07 | -0.06 | CONTIG19_10080FS000088572 | 0.36 | 0.052929352 |
| 10080 | 119404 | 119467 | 0.07 | 0.36 | -0.43 | -0.26 | CONTIG19_10080FS000098147 | 0.56 | 0.06795004 |
| 10080 | 127263 | 127320 | -0.11 | -0.06 | -0.39 | -0.34 | CONTIG19_10080FS000106006 | 0.28 | 0 |
| 10080 | 151175 | 151225 | -0.17 | 0.05 | -0.53 | -0.29 | CONTIG19_10080RS000129918 | 0.35 | 0.018184189 |
| 10137 | 206841 | 206890 | 0.07 | -0.06 | -0.56 | -0.55 | CONTIG19_10137FS000009809 | 0.56 | 0.079166848 |
| 10137 | 206879 | 206936 | -0.11 | 0.02 | -0.52 | -0.33 | CONTIG19_10137RS000009847 | 0.38 | 0.050155427 |
| 10137 | 211127 | 211180 | 0.4 | 0.28 | 0.09 | -0.04 | CONTIG19_10137RS000014095 | 0.315 | 0.010104227 |
| 10137 | 230536 | 230592 | 0.54 | 0.44 | 0.3 | 0.24 | CONTIG19_10137RS000033504 | 0.22 | 0.057715877 |
| 10137 | 233643 | 233692 | 0.26 | 0.39 | -0.23 | -0.26 | CONTIG19_10137FS000036611 | 0.57 | 0.088770297 |
| 10137 | 292843 | 292894 | 0.57 | 0.44 | 0.23 | -0.01 | CONTIG19_10137RS000095811 | 0.395 | 0.088076963 |
| 10137 | 294807 | 294867 | 0.58 | 0.42 | 0.18 | 0.11 | CONTIG19_10137FS000097775 | 0.355 | 0.080270174 |
| 10137 | 303924 | 303986 | 0.76 | 0.24 | 0.41 | -0.04 | CONTIG19_10137FS000106892 | 0.315 | 0.070446575 |
| 10198 | 342315 | 342364 | -0.88 | -0.98 | -0.47 | -0.45 | CONTIG19_10198RS000027493 | -0.47 | 0.080833388 |
| 10170 | 373703 | 373761 | 0.09 | -0.1 | -0.29 | -0.53 | CONTIG19_10170RS000001864 | 0.405 | 0.039247718 |
| 10170 | 379244 | 379293 | 0.75 | 0.81 | 0.26 | 0.36 | CONTIG19_10170RS000007405 | 0.47 | 0.027073869 |
| 10170 | 395993 | 396049 | -0.31 | -0.22 | -0.53 | -0.48 | CONTIG19_10170RS000024154 | 0.24 | 0.052929352 |
| 10170 | 396756 | 396812 | 0.1 | 0.24 | -0.54 | -0.25 | CONTIG19_10170FS000024917 | 0.565 | 0.084015871 |
| 10170 | 400887 | 400940 | 0.61 | 0.44 | 0.36 | 0.12 | CONTIG19_10170RS000029048 | 0.285 | 0.077791862 |
| 10170 | 402871 | 402928 | 0.25 | 0.18 | -0.1 | -0.2 | CONTIG19_10170FS000031032 | 0.365 | 0.026147743 |
| 10170 | 406721 | 406770 | 0.8 | 0.91 | 0.37 | 0.43 | CONTIG19_10170RS000034882 | 0.455 | 0.034943972 |
| 10170 | 407520 | 407570 | 0.08 | 0.25 | -0.26 | -0.2 | CONTIG19_10170RS000035681 | 0.395 | 0.088076963 |
| 10170 | 409094 | 409149 | 0.58 | 0.86 | 0.11 | 0.25 | CONTIG19_10170RS000037255 | 0.54 | 0.082067146 |
| 10170 | 410482 | 410550 | 0.62 | 0.61 | 0.36 | 0.4 | CONTIG19_10170RS000038643 | 0.235 | 0.067471738 |
| 10170 | 416133 | 416195 | 0.91 | 0.79 | 0.44 | 0.36 | CONTIG19_10170FS000044294 | 0.45 | 0.028275604 |
| 10170 | 442126 | 442183 | -1.36 | -1.64 | 0.17 | -0.05 | CONTIG19_10170RS000070287 | -1.56 | 0.012241179 |
| 10170 | 443062 | 443119 | -1.64 | -2.01 | 0.48 | 0.19 | CONTIG19_10170RS000071223 | -2.16 | 0.011787908 |
| 10170 | 443098 | 443153 | -1.57 | -1.72 | 0.77 | 0.22 | CONTIG19_10170FS000071259 | -2.14 | 0.059324853 |
| 10170 | 443139 | 443200 | -1.26 | -1.66 | 0.61 | 0.52 | CONTIG19_10170RS000071300 | -2.025 | 0.048634089 |
| 10170 | 443175 | 443224 | -1.49 | -1.6 | 0.49 | 0.62 | CONTIG19_10170FS000071336 | -2.1 | 0.036338755 |
| 10170 | 443206 | 443257 | -1.53 | -1.86 | 0.57 | 0.33 | CONTIG19_10170RS000071367 | -2.145 | 0.013353701 |
| 10170 | 443246 | 443304 | -1.47 | -1.8 | 0.57 | 0.38 | CONTIG19_10170FS000071407 | -2.11 | 0.021112344 |
| 10170 | 443278 | 443334 | -1.25 | -1.54 | 0.8 | 0.27 | CONTIG19_10170RS000071439 | -1.93 | 0.039531687 |
| 10170 | 443322 | 443376 | -1.32 | -1.43 | 0.77 | 0.32 | CONTIG19_10170FS000071483 | -1.92 | 0.056220765 |
| 10170 | 443350 | 443404 | -1.19 | -1.98 | 0.77 | 0.36 | CONTIG19_10170RS000071511 | -2.15 | 0.056113649 |
| 10170 | 443396 | 443452 | -1.21 | -1.85 | 0.61 | 0.15 | CONTIG19_10170FS000071557 | -1.91 | 0.029975618 |
| 10170 | 443422 | 443478 | -1.39 | -1.52 | 0.74 | 0.09 | CONTIG19_10170RS000071583 | -1.87 | 0.087950138 |
| 10170 | 443458 | 443516 | -1.58 | -1.61 | 0.72 | 0.23 | CONTIG19_10170FS000071619 | -2.07 | 0.070446575 |
| 10170 | 443504 | 443560 | -0.97 | -1.69 | 0.63 | 0.28 | CONTIG19_10170RS000071665 | -1.785 | 0.065745468 |
| 10170 | 443538 | 443589 | -1.21 | -1.96 | 0.64 | 0.42 | CONTIG19_10170FS000071699 | -2.115 | 0.079352074 |
| 10170 | 443566 | 443623 | -1.46 | -1.7 | 0.64 | 0.41 | CONTIG19_10170RS000071727 | -2.105 | 0.001512158 |
| 10170 | 443608 | 443663 | -1.53 | -2.14 | 0.38 | 0.35 | CONTIG19_10170FS000071769 | -2.2 | 0.083437012 |
| 10170 | 443648 | 443702 | -1.58 | -2 | 0.48 | 0.39 | CONTIG19_10170RS000071809 | -2.225 | 0.047123749 |
| 10170 | 443674 | 443726 | -1.49 | -1.93 | 0.3 | 0.37 | CONTIG19_10170FS000071835 | -2.045 | 0.078975268 |
| 10170 | 443712 | 443762 | -1.77 | -2.08 | 0.22 | -0.05 | CONTIG19_10170RS000071873 | -2.01 | 0.006334316 |
| 10170 | 443752 | 443809 | -1.62 | -1.96 | 0.33 | 0.41 | CONTIG19_10170FS000071913 | -2.16 | 0.061699678 |
| 10170 | 443792 | 443852 | -1.67 | -2.08 | 0.5 | 0.47 | CONTIG19_10170RS000071953 | -2.36 | 0.051142981 |
| 10170 | 443818 | 443874 | -1.72 | -1.95 | 0.49 | 0.5 | CONTIG19_10170FS000071979 | -2.33 | 0.032758341 |
| 10170 | 443855 | 443915 | -1.36 | -1.75 | 0.69 | 0.46 | CONTIG19_10170RS000072016 | -2.13 | 0.023899368 |
| 10170 | 444004 | 444061 | -1.22 | -1.69 | 0.75 | 0.52 | CONTIG19_10170RS000072165 | -2.09 | 0.036512244 |
| 10170 | 444035 | 444084 | -1.03 | -1.85 | 0.88 | 0.48 | CONTIG19_10170FS000072196 | -2.12 | 0.062856341 |
| 10170 | 444077 | 444139 | -1.33 | -2.24 | 0.83 | 0.51 | CONTIG19_10170RS000072238 | -2.455 | 0.076133068 |
| 10170 | 444106 | 444162 | -1.05 | -1.68 | 0.88 | 0.49 | CONTIG19_10170FS000072267 | -2.05 | 0.037223071 |
| 10170 | 444142 | 444201 | -1.69 | -1.86 | 0.7 | 0.32 | CONTIG19_10170RS000072303 | -2.285 | 0.029233298 |
| 10170 | 444178 | 444232 | -1.33 | -1.93 | 0.48 | 0.29 | CONTIG19_10170FS000072339 | -2.015 | 0.064545688 |
| 10170 | 444217 | 444273 | -1.06 | -2.01 | 0.91 | 0.51 | CONTIG19_10170RS000072378 | -2.245 | 0.077595813 |
| 10170 | 444250 | 444306 | -1.42 | -1.84 | 0.68 | 0.41 | CONTIG19_10170FS000072411 | -2.175 | 0.021943711 |
| 10170 | 444286 | 444335 | -1.55 | -1.55 | 0.62 | 0.49 | CONTIG19_10170RS000072447 | -2.105 | 0.019651848 |
| 10170 | 444322 | 444378 | -1.4 | -1.74 | 0.57 | 0.47 | CONTIG19_10170FS000072483 | -2.09 | 0.036512244 |
| 10170 | 444368 | 444427 | -1.25 | -1.96 | 0.84 | 0.39 | CONTIG19_10170RS000072529 | -2.22 | 0.037237012 |
| 10170 | 444402 | 444458 | -1.31 | -1.56 | 0.53 | 0.41 | CONTIG19_10170FS000072563 | -1.905 | 0.021713511 |
| 10170 | 444430 | 444482 | -1.31 | -1.56 | 0.42 | 0.18 | CONTIG19_10170RS000072591 | -1.735 | 0.001834634 |
| 10170 | 444466 | 444518 | -1.58 | -1.96 | 0.48 | -0.04 | CONTIG19_10170FS000072627 | -1.99 | 0.022384431 |
| 10170 | 444511 | 444571 | -1.43 | -1.98 | 0.35 | 0.16 | CONTIG19_10170RS000072672 | -1.96 | 0.058301543 |
| 10170 | 444723 | 444781 | -1.12 | -1.52 | 0.52 | 0.19 | CONTIG19_10170RS000072884 | -1.675 | 0.013300567 |
| 10170 | 444757 | 444815 | -1.33 | -1.71 | 0.45 | 0.32 | CONTIG19_10170FS000072918 | -1.905 | 0.041713153 |
| 10170 | 444800 | 444855 | -1.44 | -1.79 | 0.4 | 0.36 | CONTIG19_10170RS000072961 | -1.995 | 0.049362522 |
| 10170 | 445402 | 445461 | -1.42 | -1.89 | 0.12 | 0.05 | CONTIG19_10170FS000073563 | -1.74 | 0.072854961 |
| 10170 | 445916 | 445974 | -1.71 | -2.04 | 0.09 | 0.01 | CONTIG19_10170FS000074077 | -1.925 | 0.04128099 |
| 10170 | 445946 | 446005 | -1.26 | -1.78 | 0.04 | -0.09 | CONTIG19_10170RS000074107 | -1.495 | 0.0825712 |
| 10170 | 446159 | 446216 | -1.57 | -1.85 | -0.13 | -0.21 | CONTIG19_10170RS000074320 | -1.54 | 0.04128099 |
| 10170 | 446485 | 446543 | -1.74 | -2.04 | 0.05 | -0.02 | CONTIG19_10170FS000074646 | -1.905 | 0.038384533 |
| 10170 | 446519 | 446573 | -1.55 | -1.84 | 0.07 | 0.06 | CONTIG19_10170RS000074680 | -1.76 | 0.050533805 |
| 10170 | 446564 | 446626 | -1.45 | -1.92 | 0.23 | 0.39 | CONTIG19_10170FS000074725 | -1.995 | 0.099695851 |
| 10170 | 446592 | 446649 | -1.39 | -2 | 0.22 | 0.18 | CONTIG19_10170RS000074753 | -1.895 | 0.095032686 |
| 10170 | 446635 | 446691 | -1.65 | -1.93 | 0.13 | 0.01 | CONTIG19_10170FS000074796 | -1.86 | 0.02736463 |
| 10170 | 446770 | 446819 | -1.43 | -1.82 | 0 | 0.05 | CONTIG19_10170FS000074931 | -1.65 | 0.084384926 |
| 10170 | 446812 | 446861 | -1.1 | -1.58 | 0.01 | -0.14 | CONTIG19_10170RS000074973 | -1.275 | 0.081930737 |
| 10170 | 446844 | 446893 | -1.7 | -1.86 | -0.12 | -0.1 | CONTIG19_10170FS000075005 | -1.67 | 0.034275693 |
| 10170 | 446879 | 446936 | -1.43 | -1.79 | -0.09 | -0.03 | CONTIG19_10170RS000075040 | -1.55 | 0.085729706 |
| 10170 | 446920 | 446971 | -1.61 | -1.82 | -0.08 | -0.06 | CONTIG19_10170FS000075081 | -1.645 | 0.044433043 |
| 10170 | 446950 | 447001 | -1.83 | -1.92 | -0.39 | -0.29 | CONTIG19_10170RS000075111 | -1.535 | 0.039349732 |
| 10170 | 446996 | 447051 | -1.48 | -1.95 | -0.12 | -0.11 | CONTIG19_10170FS000075157 | -1.6 | 0.094786285 |
| 10170 | 447030 | 447089 | -1.72 | -1.79 | 0.02 | -0.11 | CONTIG19_10170RS000075191 | -1.71 | 0.011167622 |
| 10170 | 447061 | 447124 | -1.66 | -2.02 | -0.09 | -0.05 | CONTIG19_10170FS000075222 | -1.77 | 0.071630615 |
| 10170 | 447893 | 447955 | -1.2 | -1.27 | -0.04 | -0.11 | CONTIG19_10170RS000076054 | -1.16 | 0 |
| 10170 | 447922 | 447980 | -1.52 | -1.82 | -0.08 | -0.29 | CONTIG19_10170FS000076083 | -1.485 | 0.019285607 |
| 10170 | 447968 | 448022 | -1.88 | -1.94 | -0.15 | -0.15 | CONTIG19_10170RS000076129 | -1.76 | 0.010850423 |
| 10170 | 448004 | 448060 | -1.09 | -1.69 | 0.06 | -0.11 | CONTIG19_10170FS000076165 | -1.365 | 0.09945634 |
| 10170 | 448033 | 448082 | -1.38 | -1.38 | -0.06 | 0.18 | CONTIG19_10170RS000076194 | -1.44 | 0.052929352 |
| 10170 | 448071 | 448129 | -1.26 | -1.37 | 0.15 | -0.14 | CONTIG19_10170FS000076232 | -1.32 | 0.043338819 |
| 10170 | 448102 | 448155 | -1.21 | -1.37 | 0.18 | -0.18 | CONTIG19_10170RS000076263 | -1.29 | 0.049251872 |
| 10170 | 448147 | 448200 | -1.55 | -1.84 | 0.19 | 0.01 | CONTIG19_10170FS000076308 | -1.795 | 0.019500354 |
| 10170 | 448174 | 448230 | -1.46 | -1.74 | 0.11 | -0.02 | CONTIG19_10170RS000076335 | -1.645 | 0.029005131 |
| 10170 | 448211 | 448275 | -1.46 | -1.63 | 0.21 | -0.14 | CONTIG19_10170FS000076372 | -1.58 | 0.036224007 |
| 10170 | 448255 | 448318 | -1.45 | -1.86 | 0.35 | 0.08 | CONTIG19_10170RS000076416 | -1.87 | 0.023819565 |
| 10170 | 449075 | 449134 | -1.79 | -1.85 | 0.16 | 0.27 | CONTIG19_10170FS000077236 | -2.035 | 0.02657555 |
| 10170 | 449116 | 449173 | -1.56 | -2.03 | 0.12 | 0.14 | CONTIG19_10170RS000077277 | -1.925 | 0.080591051 |
| 10170 | 449256 | 449316 | -1.49 | -1.84 | 0.38 | 0.3 | CONTIG19_10170RS000077417 | -2.005 | 0.042800072 |
| 10170 | 449290 | 449349 | -1.63 | -1.9 | 0.43 | 0.29 | CONTIG19_10170FS000077451 | -2.125 | 0.019467006 |
| 10170 | 449329 | 449387 | -1.73 | -1.95 | 0.35 | 0.13 | CONTIG19_10170RS000077490 | -2.08 | 0 |
| 10170 | 449368 | 449420 | -1.36 | -1.89 | 0.42 | 0.34 | CONTIG19_10170FS000077529 | -2.005 | 0.071143476 |
| 10170 | 449403 | 449455 | -1.43 | -1.98 | 0.45 | 0.17 | CONTIG19_10170RS000077564 | -2.015 | 0.0425883 |
| 10170 | 449435 | 449492 | -1.23 | -1.77 | 0.49 | 0.25 | CONTIG19_10170FS000077596 | -1.87 | 0.050956654 |
| 10170 | 449471 | 449528 | -1.45 | -1.63 | 0.29 | 0.29 | CONTIG19_10170RS000077632 | -1.83 | 0.031283963 |
| 10202 | 485886 | 485935 | 0.2 | 0.31 | -0.28 | -0.35 | CONTIG19_10202RS000012508 | 0.57 | 0.099695851 |
| 10202 | 485923 | 485973 | 0.24 | 0.52 | -0.37 | -0.23 | CONTIG19_10202FS000012545 | 0.68 | 0.065304362 |
| 10202 | 485954 | 486017 | 0.36 | 0.32 | -0.07 | -0.16 | CONTIG19_10202RS000012576 | 0.455 | 0.034943972 |
| 10202 | 494254 | 494307 | -1.35 | -1.46 | -0.17 | -0.41 | CONTIG19_10202RS000020876 | -1.115 | 0.037070408 |
| 10202 | 494298 | 494356 | -1.69 | -2 | -0.12 | -0.38 | CONTIG19_10202FS000020920 | -1.595 | 0.009977549 |
| 10202 | 494519 | 494568 | -1.39 | -1.99 | -0.57 | -0.91 | CONTIG19_10202FS000021141 | -0.95 | 0.086578645 |
| 10202 | 494552 | 494607 | -1.62 | -1.86 | -0.39 | -0.18 | CONTIG19_10202RS000021174 | -1.455 | 0.097672702 |
| 10202 | 494595 | 494649 | -1.69 | -1.87 | -0.38 | -0.13 | CONTIG19_10202FS000021217 | -1.525 | 0.08916529 |
| 10202 | 494621 | 494681 | -1.85 | -2 | -0.16 | -0.1 | CONTIG19_10202RS000021243 | -1.795 | 0.037197209 |
| 10202 | 502439 | 502501 | 0.18 | 0.21 | -0.13 | -0.06 | CONTIG19_10202FS000029061 | 0.29 | 0.043835403 |
| 10202 | 502481 | 502543 | 0.25 | 0.3 | -0.14 | 0.01 | CONTIG19_10202RS000029103 | 0.34 | 0.09295429 |
| 10202 | 508785 | 508846 | 0.69 | 0.7 | 0.27 | 0.27 | CONTIG19_10202FS000035407 | 0.425 | 0.007489299 |
| 10202 | 509113 | 509172 | 0.4 | 0.45 | -0.07 | 0.03 | CONTIG19_10202RS000035735 | 0.445 | 0.0357276 |
| 10202 | 533757 | 533813 | 0.74 | 0.56 | 0.46 | 0.29 | CONTIG19_10202FS000060379 | 0.275 | 0.01157363 |
| 10202 | 538519 | 538576 | 0.21 | 0.44 | -0.11 | 0.13 | CONTIG19_10202RS000065141 | 0.315 | 0.010104227 |
| 10202 | 550977 | 551031 | 0.19 | -0.1 | -0.17 | -0.48 | CONTIG19_10202RS000077599 | 0.37 | 0.017201752 |
| 10202 | 551011 | 551070 | 0.14 | -0.03 | -0.24 | -0.46 | CONTIG19_10202FS000077633 | 0.405 | 0.039247718 |
| 10202 | 557955 | 558009 | 0.09 | 0.21 | -0.44 | -0.29 | CONTIG19_10202FS000084577 | 0.515 | 0.018537083 |
| 10202 | 557984 | 558042 | 0.21 | 0.16 | -0.15 | -0.13 | CONTIG19_10202RS000084606 | 0.325 | 0.068295841 |
| 10202 | 581488 | 581550 | 0.98 | 0.8 | 0.42 | 0.35 | CONTIG19_10202FS000108110 | 0.505 | 0.069062621 |
| 10202 | 581523 | 581579 | 0.9 | 0.65 | 0.47 | 0.29 | CONTIG19_10202RS000108145 | 0.395 | 0.056262409 |
| 10202 | 604520 | 604569 | 0.37 | 0.73 | -0.05 | 0.37 | CONTIG19_10202FS000131142 | 0.39 | 0.048874504 |
| 10202 | 607678 | 607742 | 0.56 | 0.66 | 0.03 | 0.21 | CONTIG19_10202FS000134300 | 0.49 | 0.051853982 |
| 10202 | 607713 | 607774 | 0.44 | 0.55 | -0.01 | -0.06 | CONTIG19_10202RS000134335 | 0.53 | 0.095373572 |
| 10202 | 612106 | 612155 | 0.84 | 1.05 | 0.29 | 0.4 | CONTIG19_10202RS000138728 | 0.6 | 0.052929352 |
| 10202 | 672701 | 672757 | 0.56 | 0.64 | 0.32 | 0.45 | CONTIG19_10202FS000199323 | 0.215 | 0.073694607 |
| 10202 | 672747 | 672802 | 0.84 | 0.79 | 0.53 | 0.54 | CONTIG19_10202RS000199369 | 0.28 | 0.06795004 |
| 10202 | 702113 | 702172 | 0.01 | 0.04 | -0.49 | -0.49 | CONTIG19_10202FS000228735 | 0.515 | 0.018537083 |
| 10155 | 726377 | 726426 | -0.13 | 0.02 | -0.56 | -0.34 | CONTIG19_10155FS000000292 | 0.395 | 0.056262409 |
| 10155 | 745556 | 745605 | 0.19 | 0.47 | -0.08 | 0.12 | CONTIG19_10155FS000019471 | 0.31 | 0.081693104 |
| 10155 | 759156 | 759217 | 0.03 | 0.02 | -0.29 | -0.24 | CONTIG19_10155RS000033071 | 0.29 | 0.06562379 |
| 10155 | 766673 | 766722 | 0.02 | -0.01 | -0.43 | -0.5 | CONTIG19_10155FS000040588 | 0.47 | 0.027073869 |
| 10155 | 771555 | 771607 | 0.28 | 0.24 | 0.02 | -0.06 | CONTIG19_10155FS000045470 | 0.28 | 0.045395742 |
| 2472 | 779714 | 779763 | 0.1 | 0.21 | -0.16 | -0.03 | CONTIG19_2472RS000000398 | 0.25 | 0.025451223 |
| 2472 | 781837 | 781893 | 0.9 | 0.98 | 0.52 | 0.62 | CONTIG19_2472FS000002521 | 0.37 | 0.017201752 |
| 2472 | 806125 | 806174 | 0.86 | 0.84 | 0.43 | 0.3 | CONTIG19_2472RS000026809 | 0.485 | 0.071886889 |
| 10093 | 838838 | 838887 | 0.38 | 0.2 | 0.11 | -0.09 | CONTIG19_10093RS000016825 | 0.28 | 0.022726761 |
| 10171 | 871318 | 871377 | 0.08 | 0.26 | -0.34 | -0.08 | CONTIG19_10171FS000012842 | 0.38 | 0.066766733 |
| 10171 | 871355 | 871411 | -0.09 | 0.1 | -0.37 | -0.12 | CONTIG19_10171RS000012879 | 0.25 | 0.076030816 |
| 10171 | 871388 | 871445 | -0.01 | 0.14 | -0.34 | -0.2 | CONTIG19_10171FS000012912 | 0.335 | 0.009501082 |
| 10171 | 871426 | 871487 | -0.05 | 0.08 | -0.35 | -0.22 | CONTIG19_10171RS000012950 | 0.3 | 0 |
| 10171 | 871793 | 871842 | 0.34 | 0.35 | -0.16 | -0.08 | CONTIG19_10171RS000013317 | 0.465 | 0.047827433 |
| 10194 | 928759 | 928809 | 0.79 | 0.82 | 0.16 | 0.23 | CONTIG19_10194FS000011329 | 0.61 | 0.020865305 |
| 10194 | 928786 | 928835 | 0.71 | 0.85 | 0.18 | 0.29 | CONTIG19_10194RS000011356 | 0.545 | 0.017517223 |
| 10194 | 932191 | 932247 | -0.01 | 0.32 | -0.25 | 0 | CONTIG19_10194RS000014761 | 0.28 | 0.090334471 |
| 10194 | 976004 | 976053 | 0.24 | 0.46 | -0.12 | -0.01 | CONTIG19_10194FS000058574 | 0.415 | 0.083882464 |
| 10194 | 986767 | 986823 | 0.68 | 0.85 | 0.07 | 0.21 | CONTIG19_10194FS000069337 | 0.625 | 0.015275942 |
| 10194 | 994901 | 994958 | -0.13 | -0.04 | -0.39 | -0.26 | CONTIG19_10194RS000077471 | 0.24 | 0.052929352 |
| 10194 | 998438 | 998487 | -0.18 | -0.28 | -0.45 | -0.48 | CONTIG19_10194FS000081008 | 0.235 | 0.094123829 |
| 10194 | 1018462 | 1018519 | 0.35 | 0.24 | -0.03 | -0.05 | CONTIG19_10194RS000101032 | 0.335 | 0.085007233 |
| 10194 | 1027795 | 1027850 | 0.02 | 0.02 | -0.42 | -0.3 | CONTIG19_10194FS000110365 | 0.38 | 0.099695851 |
| 10194 | 1061669 | 1061718 | 0.77 | 0.52 | 0.12 | 0.03 | CONTIG19_10194FS000144239 | 0.57 | 0.088770297 |
| 10194 | 1078489 | 1078548 | -0.05 | 0.07 | -0.48 | -0.26 | CONTIG19_10194RS000161059 | 0.38 | 0.083287307 |
| 10194 | 1103719 | 1103779 | 0.19 | 0.26 | -0.28 | -0.22 | CONTIG19_10194FS000186289 | 0.475 | 0.006701013 |
| 10194 | 1124447 | 1124505 | 0.2 | 0.1 | -0.02 | -0.09 | CONTIG19_10194FS000207017 | 0.205 | 0.046499068 |
| 10194 | 1160431 | 1160481 | 0.29 | 0.37 | -0.17 | -0.01 | CONTIG19_10194FS000243001 | 0.42 | 0.060448134 |

Ch6 – H4 acetylation

| Contig | Probe sequence start | Probe sequence end | Log2 value of Sor125(55) mutant 1 | Log2 value of Sor125(55) mutant 2 | Log2 value of 3153A parent 1 | Log2 value of 3153A parent 2 | Probe Name | Mean difference | p value |
| --- | --- | --- | --- | --- | --- | --- | --- | --- | --- |
| 10185 | 150332 | 150381 | -0.11 | -0.1 | 0.24 | 0.16 | CONTIG19_10185RS000028582 | -0.305 | 0.093254724 |
| 10185 | 150368 | 150423 | -0.08 | -0.1 | 0.24 | 0.15 | CONTIG19_10185FS000028618 | -0.285 | 0.077791862 |
| 10185 | 158729 | 158778 | 0.11 | 0.4 | -0.26 | -0.09 | CONTIG19_10185RS000036979 | 0.43 | 0.088260798 |
| 10035 | 174885 | 174934 | 0.12 | 0.28 | -0.26 | -0.12 | CONTIG19_10035FS000012269 | 0.39 | 0.016320008 |
| 10035 | 176145 | 176203 | -0.29 | -0.5 | -0.04 | -0.21 | CONTIG19_10035RS000013529 | -0.27 | 0.047071053 |
| 10035 | 210963 | 211020 | -0.28 | -0.25 | 0.13 | 0.19 | CONTIG19_10035RS000048347 | -0.425 | 0.02245961 |
| 10035 | 217023 | 217072 | 0.33 | 0.47 | 0.01 | 0.03 | CONTIG19_10035RS000054407 | 0.38 | 0.099695851 |
| 10181 | 426380 | 426442 | 0.22 | 0.44 | -0.17 | 0.02 | CONTIG19_10181FS000009905 | 0.405 | 0.023567738 |
| 10181 | 426414 | 426477 | 0.27 | 0.38 | -0.26 | -0.17 | CONTIG19_10181RS000009939 | 0.54 | 0.011787908 |
| 10230 | 565410 | 565466 | 0.56 | 0.52 | 0.1 | 0.09 | CONTIG19_10230RS000060782 | 0.445 | 0.021450972 |
| 10230 | 604055 | 604117 | 0.14 | 0.16 | -0.17 | -0.11 | CONTIG19_10230FS000099427 | 0.29 | 0.043835403 |
| 10230 | 604083 | 604132 | 0.16 | 0.71 | -0.42 | 0.11 | CONTIG19_10230RS000099455 | 0.59 | 0.010789133 |
| 10230 | 708620 | 708672 | -0.76 | -0.49 | -0.37 | -0.14 | CONTIG19_10230RS000203992 | -0.37 | 0.034378423 |
| 2201 | 898485 | 898546 | 0.28 | 0.01 | 0.05 | -0.19 | CONTIG19_2201FS000003459 | 0.215 | 0.044343479 |
| 10090 | 927256 | 927312 | 0.35 | 0.3 | -0.01 | 0.03 | CONTIG19_10090FS000003675 | 0.315 | 0.090334471 |
| 10090 | 967214 | 967270 | 0.11 | -0.01 | -0.13 | -0.23 | CONTIG19_10090FS000043633 | 0.23 | 0.027661699 |
| 10140 | 1014430 | 1014486 | 0.4 | 0.54 | 0.14 | 0.24 | CONTIG19_10140RS000027218 | 0.28 | 0.045395742 |
| 10140 | 1017943 | 1017992 | 0.79 | 0.67 | 0.4 | 0.28 | CONTIG19_10140FS000030731 | 0.39 | 0 |

Ch7 – H4 acetylation

| Contig | Probe sequence start | Probe sequence end | Log2 value of Sor125(55) mutant 1 | Log2 value of Sor125(55) mutant 2 | Log2 value of 3153A parent 1 | Log2 value of 3153A parent 2 | Probe Name | Mean difference | p value |
| --- | --- | --- | --- | --- | --- | --- | --- | --- | --- |
| 10262 | 188760 | 188816 | 0.33 | 0.47 | -0.01 | 0.15 | CONTIG19_10262FS000188759 | 0.33 | 0.019285607 |
| 10248 | 374237 | 374292 | 0.06 | 0.21 | -0.29 | -0.23 | CONTIG19_10248RS000085233 | 0.395 | 0.072214959 |
| 10248 | 380439 | 380499 | 0.56 | 0.73 | 0.04 | 0.24 | CONTIG19_10248RS000091435 | 0.505 | 0.01890394 |
| 10248 | 437606 | 437655 | -0.32 | 0.01 | -0.62 | -0.26 | CONTIG19_10248RS000148602 | 0.285 | 0.033475417 |
| 10248 | 463985 | 464041 | 0.14 | 0.1 | -0.23 | -0.28 | CONTIG19_10248FS000174981 | 0.375 | 0.008487761 |
| 10248 | 471065 | 471122 | 0.19 | 0.31 | -0.03 | 0.11 | CONTIG19_10248FS000182061 | 0.21 | 0.030292344 |
| 10248 | 475841 | 475898 | -0.18 | -0.34 | 0.12 | -0.08 | CONTIG19_10248FS000186837 | -0.28 | 0.045395742 |
| 10219 | 649248 | 649301 | -1.15 | -1.79 | 0.23 | 0.02 | CONTIG19_10219RS000045400 | -1.595 | 0.085299797 |
| 10219 | 649383 | 649433 | -1.4 | -1.72 | 0.29 | -0.01 | CONTIG19_10219RS000045535 | -1.7 | 0.003744779 |
| 10219 | 649424 | 649480 | -1.48 | -1.82 | 0.08 | 0.12 | CONTIG19_10219FS000045576 | -1.75 | 0.068849038 |
| 10219 | 649464 | 649520 | -1.78 | -2.21 | 0.02 | 0.12 | CONTIG19_10219RS000045616 | -2.065 | 0.081252875 |
| 10219 | 667609 | 667671 | -0.12 | 0.21 | -0.44 | -0.17 | CONTIG19_10219FS000063761 | 0.35 | 0.054434361 |
| 10219 | 667653 | 667714 | -0.13 | -0.2 | -0.48 | -0.64 | CONTIG19_10219RS000063805 | 0.395 | 0.072214959 |
| 10219 | 667691 | 667753 | -0.25 | 0.02 | -0.57 | -0.33 | CONTIG19_10219FS000063843 | 0.335 | 0.028486336 |
| 10219 | 681547 | 681597 | 0.41 | 0.49 | 0.07 | 0.05 | CONTIG19_10219RS000077699 | 0.39 | 0.081175106 |
| 10110 | 737039 | 737097 | 0.87 | 0.56 | 0.46 | 0.15 | CONTIG19_10110FS000017295 | 0.41 | 6.09483E-17 |
| 10110 | 737074 | 737130 | 0.58 | 0.46 | 0.21 | 0.03 | CONTIG19_10110RS000017330 | 0.4 | 0.047657259 |
| 2506 | 883680 | 883733 | -0.69 | -0.81 | 0 | -0.3 | CONTIG19_2506RS000045636 | -0.6 | 0.094786285 |
| 2506 | 892659 | 892708 | 0.32 | 0.48 | -0.06 | 0.02 | CONTIG19_2506FS000054615 | 0.42 | 0.060448134 |
| 2506 | 943417 | 943481 | 0.07 | 0.21 | -0.23 | -0.08 | CONTIG19_2506RS000105373 | 0.295 | 0.010789133 |

ChR – H4 acetylation

| Contig | Probe sequence start | Probe sequence end | Log2 value of Sor125(55) mutant 1 | Log2 value of Sor125(55) mutant 2 | Log2 value of 3153A parent 1 | Log2 value of 3153A parent 2 | Probe Name | Mean difference | p value |
| --- | --- | --- | --- | --- | --- | --- | --- | --- | --- |
| 2516 | 19901 | 19959 | -0.09 | -0.41 | 0.29 | 0 | CONTIG19_2516FS000019900 | -0.395 | 0.024163823 |
| 2516 | 21193 | 21252 | 0.51 | 0.34 | 0.25 | 0.14 | CONTIG19_2516FS000021192 | 0.23 | 0.0825712 |
| 2516 | 113758 | 113807 | 0.28 | 0.02 | 0.03 | -0.24 | CONTIG19_2516FS000113757 | 0.255 | 0.012481141 |
| 2516 | 137574 | 137634 | -0.22 | -0.17 | -0.59 | -0.48 | CONTIG19_2516FS000137573 | 0.34 | 0.056027234 |
| 2516 | 154439 | 154491 | -0.68 | -0.54 | -0.39 | -0.28 | CONTIG19_2516FS000154438 | -0.275 | 0.034690338 |
| 10172 | 206682 | 206731 | -0.15 | -0.16 | -0.61 | -0.54 | CONTIG19_10172RS000038765 | 0.42 | 0.060448134 |
| 10172 | 206728 | 206784 | 0.1 | 0.14 | -0.42 | -0.29 | CONTIG19_10172FS000038811 | 0.475 | 0.060131879 |
| 10172 | 210774 | 210823 | 0.27 | 0.16 | -0.05 | -0.09 | CONTIG19_10172RS000042857 | 0.285 | 0.077791862 |
| 10172 | 216171 | 216223 | 0.06 | 0.03 | -0.3 | -0.41 | CONTIG19_10172RS000048254 | 0.4 | 0.063451035 |
| 10172 | 257985 | 258052 | 0.55 | 0.43 | 0.36 | 0.21 | CONTIG19_10172FS000090068 | 0.205 | 0.046499068 |
| 10172 | 313532 | 313589 | 0.26 | 0.28 | -0.26 | -0.1 | CONTIG19_10172FS000145615 | 0.45 | 0.098242384 |
| 10172 | 317384 | 317439 | -1.49 | -1.78 | -0.15 | -0.07 | CONTIG19_10172RS000149467 | -1.525 | 0.076853747 |
| 10161 | 583384 | 583433 | 0.45 | 0.62 | 0.02 | 0.17 | CONTIG19_10161RS000006252 | 0.44 | 0.014466141 |
| 10161 | 666549 | 666617 | -0.07 | 0.17 | -0.37 | -0.19 | CONTIG19_10161FS000089417 | 0.33 | 0.057715877 |
| 10161 | 683139 | 683188 | -0.1 | 0.09 | -0.39 | -0.24 | CONTIG19_10161FS000106007 | 0.31 | 0.0410154 |
| 10161 | 683173 | 683229 | -0.18 | 0.14 | -0.42 | -0.17 | CONTIG19_10161RS000106041 | 0.275 | 0.080591051 |
| 10148 | 718835 | 718891 | -0.04 | -0.08 | -0.32 | -0.38 | CONTIG19_10148RS000026560 | 0.29 | 0.021943711 |
| 10053 | 910525 | 910580 | -0.23 | -0.22 | 0.04 | 0.14 | CONTIG19_10053FS000016323 | -0.315 | 0.090334471 |
| 10053 | 958987 | 959052 | 0.34 | 0.36 | -0.05 | 0.06 | CONTIG19_10053FS000064785 | 0.345 | 0.0825712 |
| 10063 | 1090461 | 1090523 | 0.21 | 0.38 | -0.03 | 0.09 | CONTIG19_10063RS000010836 | 0.265 | 0.059881242 |
| 10063 | 1090492 | 1090541 | 0.18 | 0.23 | -0.12 | -0.02 | CONTIG19_10063FS000010867 | 0.275 | 0.057715877 |
| 10251 | 1203049 | 1203101 | -0.53 | -0.59 | -0.15 | -0.24 | CONTIG19_10251FS000007405 | -0.365 | 0.026147743 |
| 10251 | 1228532 | 1228581 | -0.68 | -0.41 | -0.37 | -0.14 | CONTIG19_10251FS000032888 | -0.29 | 0.043835403 |
| 10251 | 1228567 | 1228618 | -0.72 | -0.31 | -0.31 | 0.15 | CONTIG19_10251RS000032923 | -0.435 | 0.036547141 |
| 20070 | 1377214 | 1377274 | 0.39 | 0.13 | 0.15 | -0.12 | CONTIG19_20070RS000030379 | 0.245 | 0.012990437 |
| 20070 | 1400023 | 1400085 | 0.35 | 0.39 | 0.14 | 0.19 | CONTIG19_20070FS000053188 | 0.205 | 0.015524234 |
| 10147 | 1499278 | 1499334 | -0.43 | -0.54 | -0.2 | -0.26 | CONTIG19_10147RS000012395 | -0.255 | 0.062214882 |
| 10147 | 1500210 | 1500265 | -0.52 | -0.48 | -0.11 | -0.12 | CONTIG19_10147RS000013327 | -0.385 | 0.04128099 |
| 10147 | 1541255 | 1541318 | -1.32 | -1.86 | 0.57 | 0.26 | CONTIG19_10147RS000054372 | -2.005 | 0.036474388 |
| 20011 | 1558129 | 1558185 | 0.23 | 0.35 | -0.29 | -0.07 | CONTIG19_20011RS000006673 | 0.47 | 0.067471738 |
| 20011 | 1558166 | 1558215 | -0.07 | 0.26 | -0.43 | -0.13 | CONTIG19_20011FS000006710 | 0.375 | 0.025451223 |
| 10057 | 1725490 | 1725539 | -1.13 | -1.38 | 0.28 | 0.06 | CONTIG19_10057FS000014400 | -1.425 | 0.006701013 |
| 10057 | 1729229 | 1729282 | 0.37 | 0.07 | 0.14 | -0.14 | CONTIG19_10057FS000018139 | 0.22 | 0.028917358 |
| 10057 | 1746053 | 1746112 | 0.34 | 0.44 | -0.1 | 0.01 | CONTIG19_10057FS000034963 | 0.435 | 0.007317146 |
| 10057 | 1751828 | 1751884 | -1.47 | -1.64 | 0.32 | 0.12 | CONTIG19_10057FS000040738 | -1.775 | 0.005379757 |
| 10057 | 1751861 | 1751922 | -2.14 | -2.01 | 0.36 | 0.08 | CONTIG19_10057RS000040771 | -2.295 | 0.056715296 |
| 10057 | 1751901 | 1751960 | -1.58 | -1.92 | 0.49 | 0.1 | CONTIG19_10057FS000040811 | -2.045 | 0.00778225 |
| 10057 | 1752278 | 1752340 | -1.15 | -1.2 | 0.1 | 0 | CONTIG19_10057FS000041188 | -1.225 | 0.012990437 |
| 10057 | 1752595 | 1752653 | -1.35 | -1.52 | 0.5 | 0.05 | CONTIG19_10057RS000041505 | -1.71 | 0.052004929 |
| 10057 | 1752634 | 1752696 | -1.81 | -2.05 | 0.25 | 0.13 | CONTIG19_10057FS000041544 | -2.12 | 0.018012732 |
| 10057 | 1752668 | 1752723 | -1.64 | -1.98 | 0.28 | 0.11 | CONTIG19_10057RS000041578 | -2.005 | 0.026972717 |
| 10057 | 1752713 | 1752769 | -1.25 | -1.73 | 0.38 | -0.09 | CONTIG19_10057FS000041623 | -1.635 | 0.001946843 |
| 10057 | 1752848 | 1752911 | -1.27 | -1.95 | 0.36 | 0.07 | CONTIG19_10057FS000041758 | -1.825 | 0.06776528 |
| 10057 | 1752883 | 1752944 | -1.47 | -1.79 | 0.33 | 0.1 | CONTIG19_10057RS000041793 | -1.845 | 0.015524234 |
| 10057 | 1752919 | 1752975 | -1.26 | -1.67 | 0.28 | -0.01 | CONTIG19_10057FS000041829 | -1.6 | 0.02386206 |
| 10057 | 1752962 | 1753019 | -1.37 | -1.42 | 0.14 | 0.03 | CONTIG19_10057RS000041872 | -1.48 | 0.012902688 |
| 10057 | 1753001 | 1753058 | -1.38 | -1.89 | 0.2 | 0.22 | CONTIG19_10057FS000041911 | -1.845 | 0.09081749 |
| 10057 | 1753217 | 1753275 | -1.4 | -1.7 | 0.02 | -0.26 | CONTIG19_10057FS000042127 | -1.43 | 0.004451814 |
| 10057 | 1753253 | 1753309 | -1.48 | -1.29 | -0.02 | -0.12 | CONTIG19_10057RS000042163 | -1.315 | 0.069915173 |
| 10057 | 1753288 | 1753343 | -1.42 | -1.8 | -0.02 | -0.31 | CONTIG19_10057FS000042198 | -1.445 | 0.019819124 |
| 10057 | 1753318 | 1753373 | -1.61 | -1.54 | 0.01 | -0.24 | CONTIG19_10057RS000042228 | -1.46 | 0.069489253 |
| 10057 | 1754971 | 1755032 | -1.37 | -1.95 | 0.13 | 0.01 | CONTIG19_10057RS000043881 | -1.73 | 0.084143874 |
| 10057 | 1755012 | 1755074 | -1.6 | -1.91 | 0.17 | -0.05 | CONTIG19_10057FS000043922 | -1.815 | 0.015780728 |
| 10057 | 1755043 | 1755104 | -1.63 | -1.84 | 0.08 | -0.12 | CONTIG19_10057RS000043953 | -1.715 | 0.001856029 |
| 10057 | 1755341 | 1755392 | -1.95 | -2.27 | -0.1 | -0.18 | CONTIG19_10057RS000044251 | -1.97 | 0.038731013 |
| 10057 | 1755370 | 1755430 | -1.66 | -1.9 | -0.16 | -0.21 | CONTIG19_10057FS000044280 | -1.595 | 0.037873049 |
| 10057 | 1755411 | 1755475 | -1.7 | -1.95 | 0 | -0.02 | CONTIG19_10057RS000044321 | -1.815 | 0.040282941 |
| 10057 | 1755629 | 1755687 | -1.69 | -1.72 | 0.19 | 0.21 | CONTIG19_10057RS000044539 | -1.905 | 0.008354111 |
| 10057 | 1755660 | 1755724 | -1.56 | -1.77 | 0.15 | 0.16 | CONTIG19_10057FS000044570 | -1.82 | 0.03843027 |
| 10057 | 1755692 | 1755755 | -1.5 | -1.39 | -0.04 | -0.02 | CONTIG19_10057RS000044602 | -1.415 | 0.020239037 |
| 10057 | 1755729 | 1755792 | -1.66 | -1.68 | 0.15 | 0.07 | CONTIG19_10057FS000044639 | -1.78 | 0.010728531 |
| 10057 | 1756562 | 1756620 | -1.27 | -1.75 | 0.2 | 0.22 | CONTIG19_10057RS000045472 | -1.72 | 0.091888462 |
| 10057 | 1756600 | 1756660 | -1.78 | -1.77 | 0.29 | 0.2 | CONTIG19_10057FS000045510 | -2.02 | 0.015754698 |
| 10247 | 1765323 | 1765379 | 0.34 | 0.28 | -0.06 | -0.09 | CONTIG19_10247FS000007206 | 0.385 | 0.024790829 |
| 10247 | 1833245 | 1833301 | 0.13 | 0.08 | 0.34 | 0.31 | CONTIG19_10247RS000075128 | -0.22 | 0.028917358 |
| 2511 | 1929755 | 1929815 | 0.05 | 0.12 | -0.15 | -0.13 | CONTIG19_2511FS000032005 | 0.225 | 0.070446575 |
| 2518 | 2090150 | 2090204 | -0.11 | 0.1 | -0.38 | -0.14 | CONTIG19_2518FS000019781 | 0.255 | 0.037405118 |
| 2518 | 2102826 | 2102883 | 0.12 | 0.08 | -0.11 | -0.18 | CONTIG19_2518RS000032457 | 0.245 | 0.038928129 |
| 2518 | 2102852 | 2102901 | 0.18 | 0.32 | -0.2 | 0.01 | CONTIG19_2518FS000032483 | 0.345 | 0.064364406 |
| 2518 | 2102888 | 2102937 | 0.18 | 0.37 | -0.16 | 0 | CONTIG19_2518RS000032519 | 0.355 | 0.026883436 |
