# Supplementary figures and images for: NuA4 histone acetyltransferase activity is required for H4 acetylation on a dosage-compensated monosomic chromosome that confers resistance to fungal toxins
Source: Epigenetics Chromatin. 2017 Oct 23;10:49. doi: 10.1186/s13072-017-0156-y (PMC5653997; doi:10.1186/s13072-017-0156-y)

## Slide 1
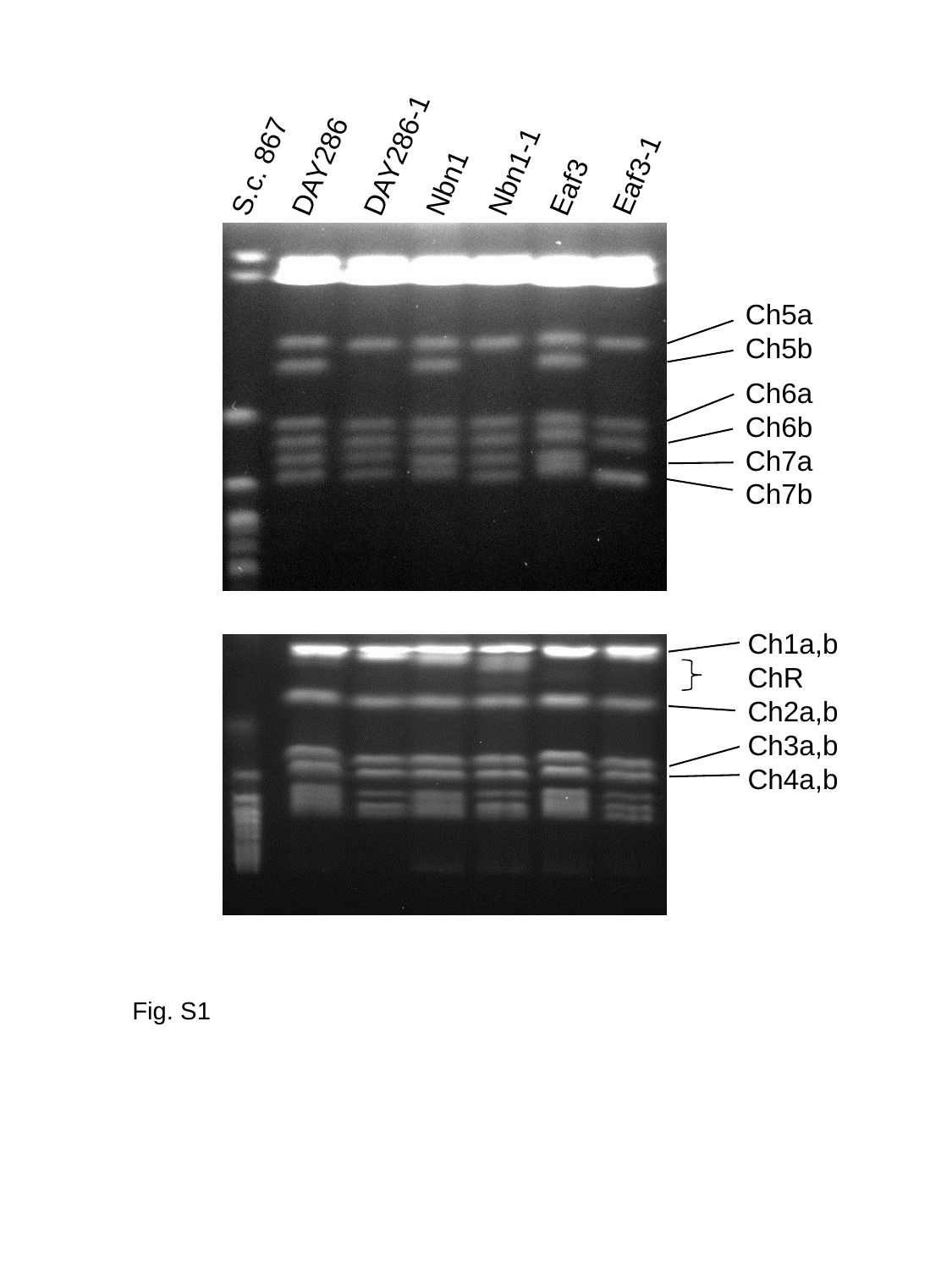

Eaf3-1
DAY286-1
Nbn1
Nbn1-1
Eaf3
DAY286
S.c. 867
Ch5a
Ch5b
Ch6a
Ch6b
Ch7a
Ch7b
Ch1a,b
ChR
Ch2a,b
Ch3a,b
Ch4a,b
Fig. S1

Supplement: Supplementary file 2 — Additional file 2: Figure S1. Chromosome separation with PFGE of C. albicans mutants that adapted to utilize toxic l-sorbose. Names of the mutants and their parental strains are indicated on a top. Top gel shows precise separation of three smallest chromosomes 7, 6, and 5, as indicated on the right, while longer chromosomes are compressed in a top portion of the gel. Note that each of these chromosomes is presented by two bands, because homologous chromosomes in each pair are not of the equal size. Bottom gel shows precise separation of chromosomes 4, 3, 2, 1, and R, as indicated on the right. Note the lack of one chromosome 5 in the mutants. Also shown are chromosomes of the Saccharomyces cerevisiae strain 867 that serve as markers of C. albicans chromosomes. [file 13072_2017_156_MOESM2_ESM.pptx]

## Slide 1
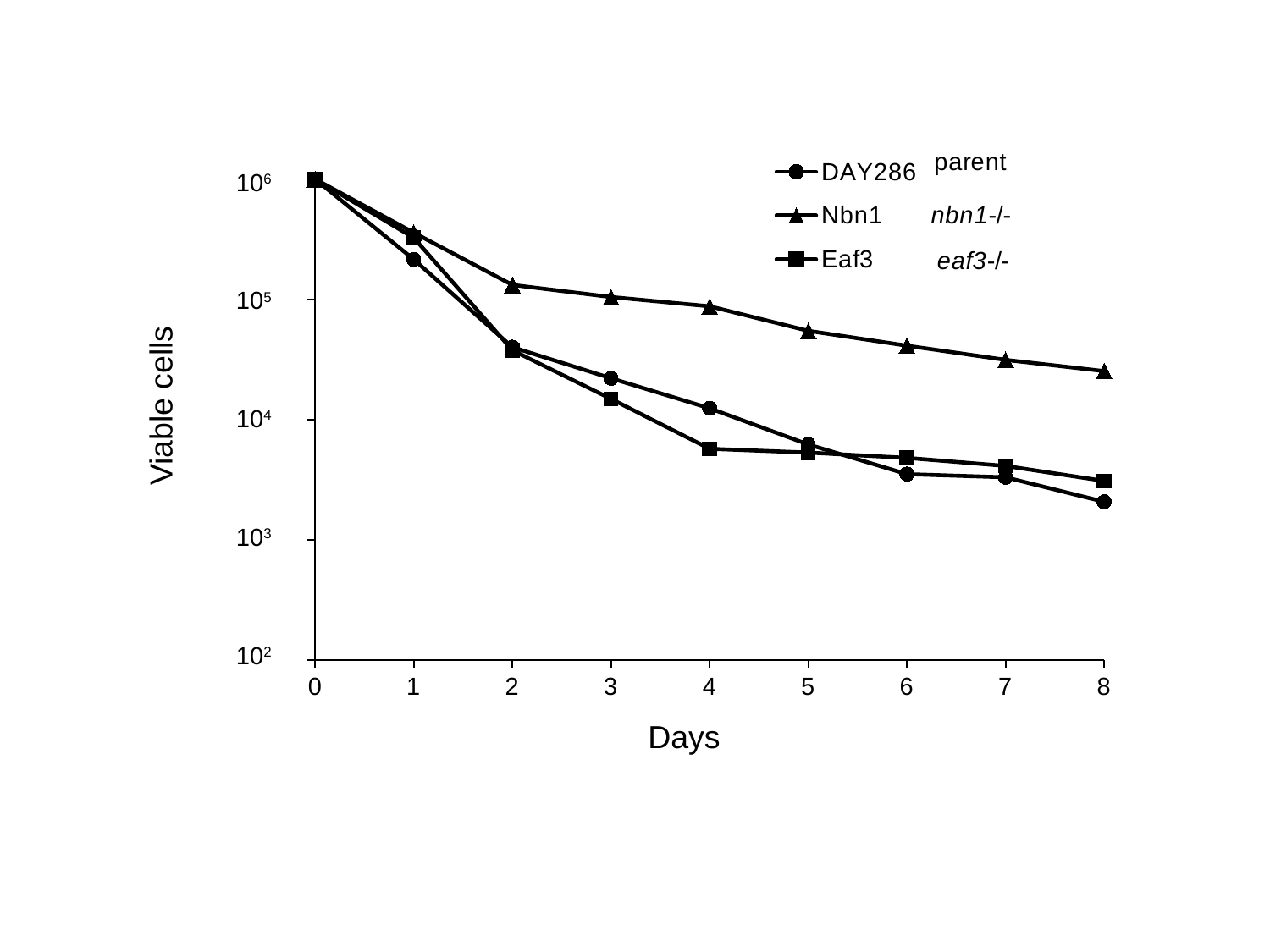

### Chart
| Category | DAY286 | Nbn1 | Eaf3 |
|---|---|---|---|106
105
104
103
102
Viable cells
Days

Supplement: Supplementary file 5 — Additional file 5: Figure S4. The survival of DAY286, Nbn1 (nbn1 −/−), and Eaf3 (eaf3 −/−) Sou− cells on l-sorbose medium. Daily survival rate was measured according to (18). [file 13072_2017_156_MOESM5_ESM.pptx]
