# Supplementary material for: NuA4 histone acetyltransferase activity is required for H4 acetylation on a dosage-compensated monosomic chromosome that confers resistance to fungal toxins
Source: Epigenetics Chromatin. 2017 Oct 23;10:49. doi: 10.1186/s13072-017-0156-y (PMC5653997; doi:10.1186/s13072-017-0156-y)
Supplement: Supplementary file 3 — Additional file 3: Figure S2. Example of Western blot with histone H4 antibodies of C. albicans strains as indicated on the top. Antibodies are indicated on the left. Purified histone extract from each strain was prepared and subjected to electrophoresis on 15% polyacrylamide gels followed by Western blot analysis (Methods). [file 13072_2017_156_MOESM3_ESM.pptx]

## Slide 1
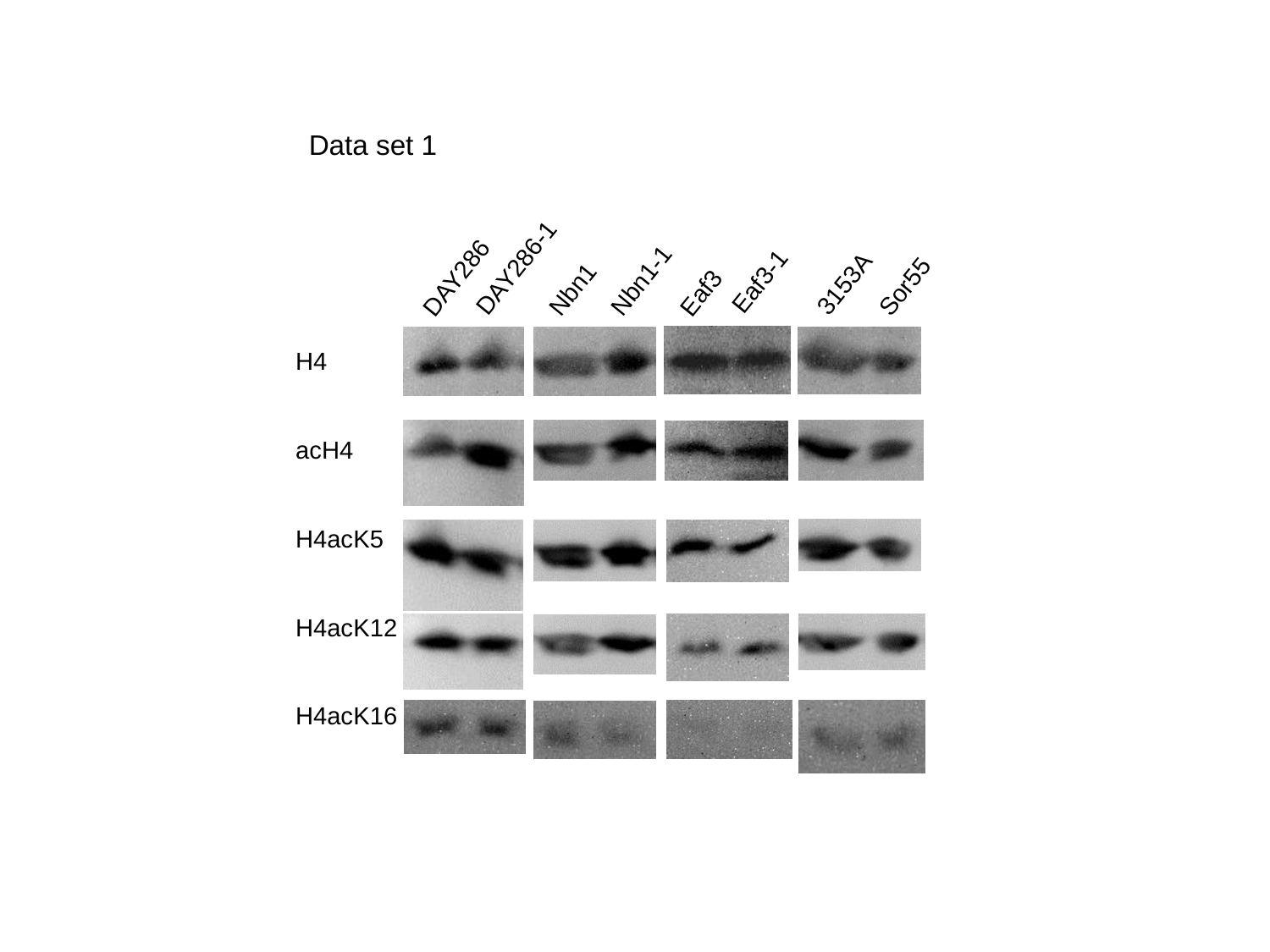

Data set 1
Eaf3-1
DAY286-1
3153A
Nbn1-1
Sor55
Nbn1
DAY286
Eaf3
H4
acH4
H4acK5
H4acK12
H4acK16

## Slide 2
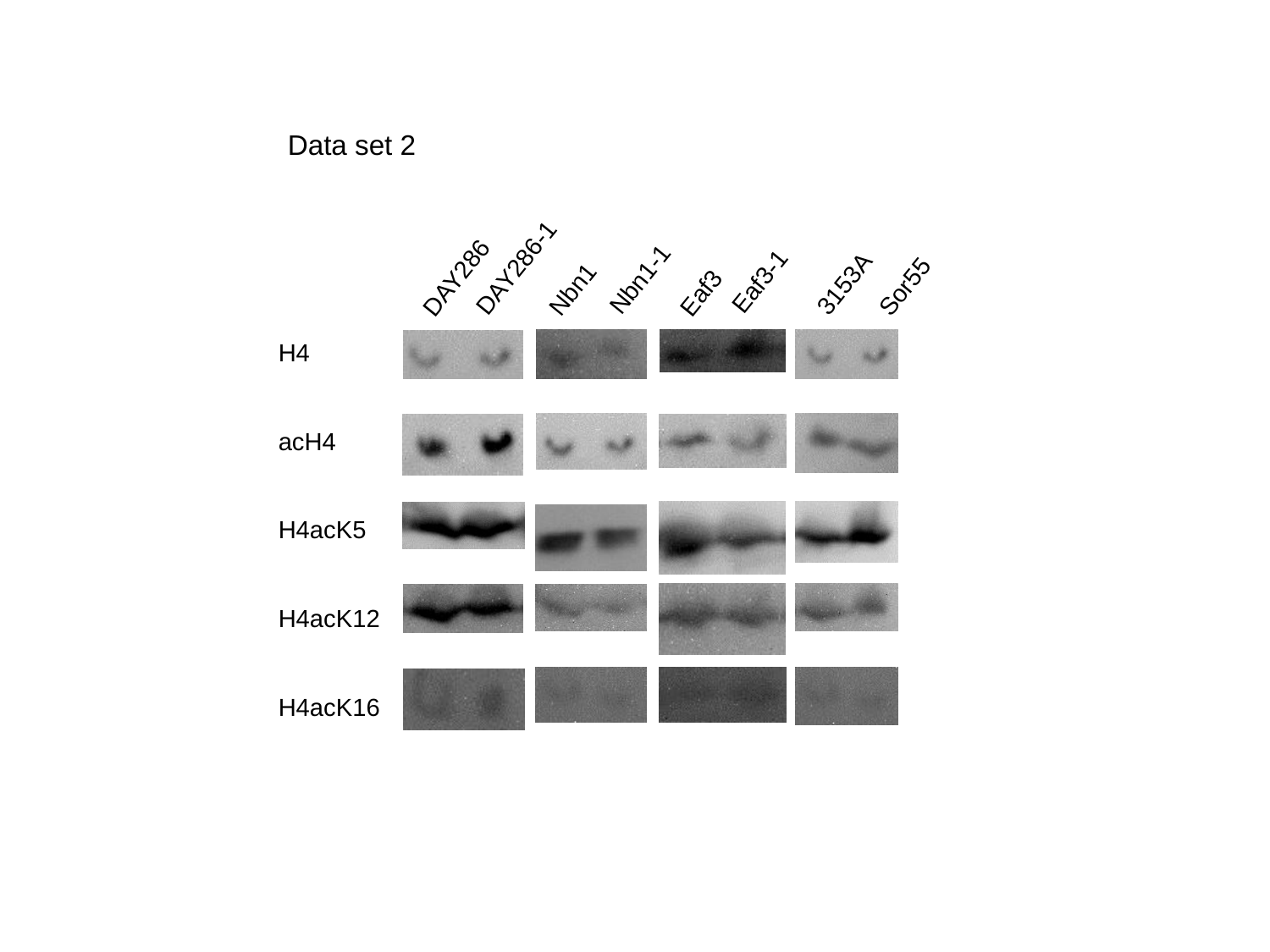

Data set 2
Nbn1-1
Eaf3-1
DAY286-1
3153A
Sor55
Nbn1
DAY286
Eaf3
H4
acH4
H4acK5
H4acK12
H4acK16

## Slide 3
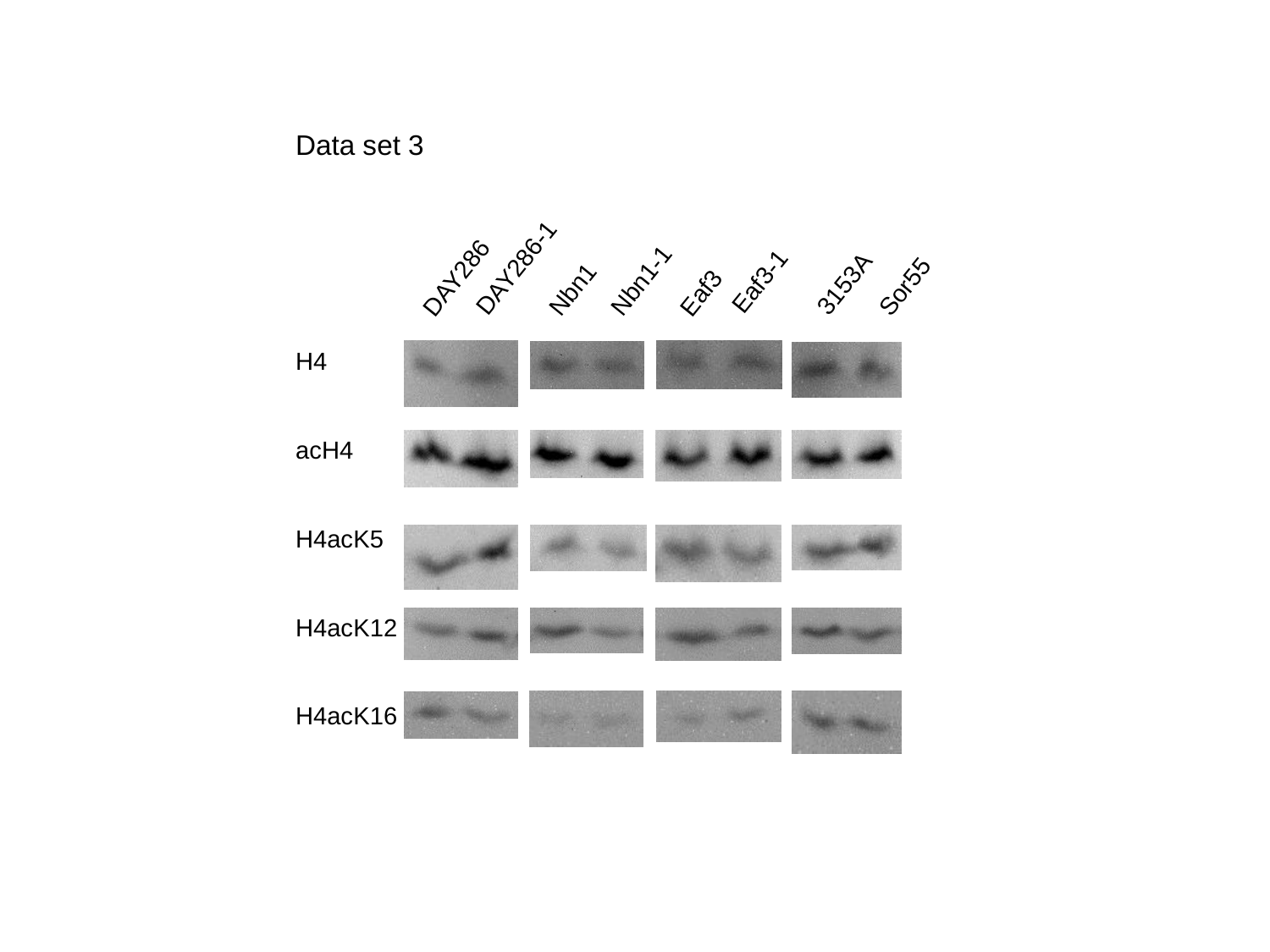

Data set 3
Eaf3-1
DAY286-1
3153A
Nbn1-1
Sor55
Nbn1
DAY286
Eaf3
H4
acH4
H4acK5
H4acK12
H4acK16
