# Supplementary material for: NuA4 histone acetyltransferase activity is required for H4 acetylation on a dosage-compensated monosomic chromosome that confers resistance to fungal toxins
Source: Epigenetics Chromatin. 2017 Oct 23;10:49. doi: 10.1186/s13072-017-0156-y (PMC5653997; doi:10.1186/s13072-017-0156-y)
Supplement: Supplementary file 4 — Additional file 4: Figure S3. Histone H3 acetylation. (A) Example of Western blot with histone H3 antibodies of C. albicans strains as indicated on the top. For details, see the legend of Fig. S2. (B) Relative amount of H3 acetylation calculated from three independent Western blot analyses. [file 13072_2017_156_MOESM4_ESM.pptx]

## Slide 1
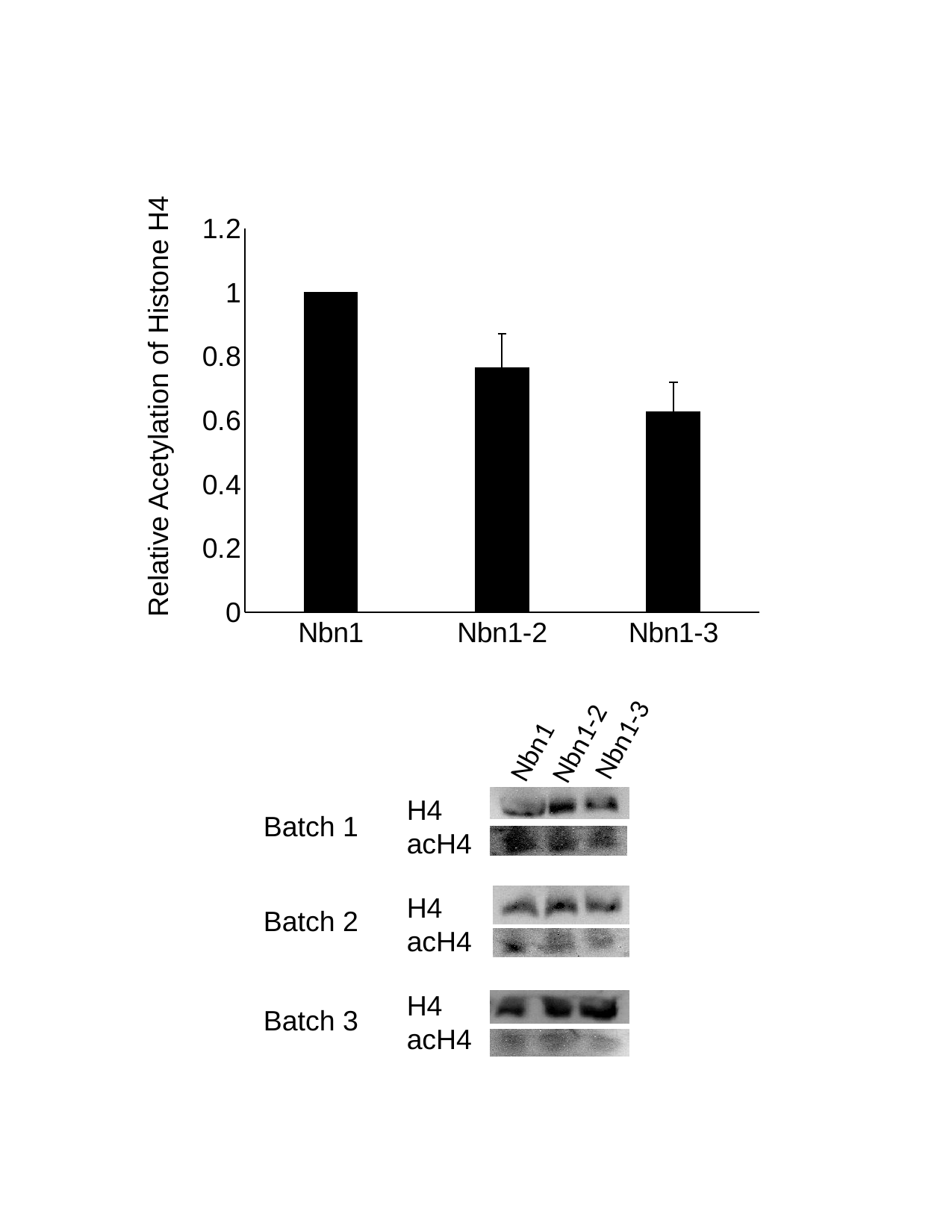

### Chart
| Category | |
|---|---|
| Nbn1 | 1.0 |
| Nbn1-2 | 0.7653514231295078 |
| Nbn1-3 | 0.6275021367054253 |Relative Acetylation of Histone H4
Nbn1-3
Nbn1-2
Nbn1
H4
acH4
Batch 1
H4
acH4
Batch 2
H4
acH4
Batch 3
